# Multi-strain Tn-Seq reveals common daptomycin resistance determinants in *Staphylococcus aureus*

**DOI:** 10.1371/journal.ppat.1007862

**Published:** 2019-11-18

**Authors:** Kathryn A. Coe, Wonsik Lee, Madeleine C. Stone, Gloria Komazin-Meredith, Timothy C. Meredith, Yonatan H. Grad, Suzanne Walker

**Affiliations:** 1 Department of Microbiology, Harvard Medical School, Boston, Massachusetts, United States of America; 2 School of Pharmacy, Sungkyunkwan University, Suwon, Republic of Korea; 3 Department of Biochemistry and Molecular Biology, Pennsylvania State University, Pennsylvania, United States of America; 4 Department of Immunology and Infectious Diseases, Harvard T.H. Chan School of Public Health, Boston, Massachusetts, United States of America; 5 Division of Infectious Diseases, Brigham and Women’s Hospital, Harvard Medical School, Boston, Massachusetts, United States of America; 6 Department of Chemistry and Chemical Biology, Harvard University, Cambridge, Massachusetts, United States of America; University of Tubingen, GERMANY

## Abstract

Antibiotic-resistant *Staphylococcus aureus* remains a leading cause of antibiotic resistance-associated mortality in the United States. Given the reality of multi-drug resistant infections, it is imperative that we establish and maintain a pipeline of new compounds to replace or supplement our current antibiotics. A first step towards this goal is to prioritize targets by identifying the genes most consistently required for survival across the *S*. *aureus* phylogeny. Here we report the first direct comparison of multiple strains of *S*. *aureus* via transposon sequencing. We show that mutant fitness varies by strain in key pathways, underscoring the importance of using more than one strain to differentiate between core and strain-dependent essential genes. We treated the libraries with daptomycin to assess whether the strain-dependent differences impact pathways important for survival. Despite baseline differences in gene importance, several pathways, including the lipoteichoic acid pathway, consistently promote survival under daptomycin exposure, suggesting core vulnerabilities that can be exploited to resensitize daptomycin-nonsusceptible isolates. We also demonstrate the merit of using transposons with outward-facing promoters capable of overexpressing nearby genes for identifying clinically-relevant gain-of-function resistance mechanisms. Together, the daptomycin vulnerabilities and resistance mechanisms support a mode of action with wide-ranging effects on the cell envelope and cell division. This work adds to a growing body of literature demonstrating the nuanced insights gained by comparing Tn-Seq results across multiple bacterial strains.

## Author summary

Antibiotic-resistant *Staphylococcus aureus* kills thousands of people every year in the United States alone. To stay ahead of the looming threat of multidrug-resistant infections, we must continue to develop new antibiotics and find ways to make our current repertoire of antibiotics more effective, including by finding pairs of compounds that perform best when administered together. In the age of next-generation sequencing, we can now use transposon sequencing to find potential targets for new antibiotics on a genome-wide scale, identified as either essential genes or genes that positively influence survival in the presence of an antibiotic. In this work, we created a compendium of genes that are essential across a range of *S*. *aureus* strains, as well as those that are important for growth in the presence of the antibiotic daptomycin. The results will be a resource for researchers working to develop the next generation of antibiotic therapies.

## Introduction

Every year in the United States over two million people acquire an antibiotic-resistant infection, and over 23,000 people die from those infections [1]. More people die of infections caused by antibiotic-resistant *Staphylococcus aureus* than any other resistant pathogen [2]. The primary antibiotic class used to treat *S*. *aureus* infections is the β-lactams, but over 50% of *S*. *aureus* infections in the United States are resistant to methicillin and other β-lactamase-impervious β-lactams [3, 4]. These β-lactam-resistant strains are termed methicillin-resistant *S*. *aureus* (MRSA). Antibiotics such as vancomycin, linezolid, and daptomycin are used to treat MRSA infections but are ineffective in an increasing number of cases [5–7]. We need a pipeline to identify new compounds that can either kill *S*. *aureus* outright or resensitize antibiotic-resistant isolates, regardless of genetic background. Thousands of *S*. *aureus* isolates have been sequenced, revealing substantial differences in gene content [8, 9], but the functional variability across the phylogeny has yet to be systematically investigated. Transposon sequencing (Tn-Seq) can be used to attribute phenotypes to genes on a genome-wide scale, enabling researchers to characterize the full essential genome of a bacterial strain in a single experiment [10, 11]. Here we describe the first multi-strain Tn-Seq study in *S*. *aureus*, using comparisons of results from favorable growth conditions to define baseline variation in genetic dependencies and then exposing the libraries to daptomycin to probe the factors that modulate daptomycin susceptibility.

Several transposon mutagenesis studies have formerly analyzed the essential genes in individual *S*. *aureus* strains [12–18]. Aside from one study conducted in a livestock-associated strain, all the studies involved one clonal complex, CC8, and most emphasized laboratory-adapted strains. Only two methicillin-resistant strains have been examined. Moreover, the conditions for library creation and analysis differed substantially. With these limitations, it is difficult to assess the congruence of gene essentiality across *S*. *aureus* strains. A recent comparative Tn-Seq analysis of *Mycobacterium tuberculosis* showed strain-to-strain differences in gene essentiality, with implications for antibiotic targets and acquisition of resistance; this work highlighted the importance of carrying out functional genomics studies of important pathogens in multiple strains using the same methodology [19]. Another comparative Tn-Seq study, performed in *Pseudomonas aeruginosa*, suggests that at least four strains need to be analyzed under comparable conditions to accurately define the core essential genome of a bacterium [20].

**Fig 1:**
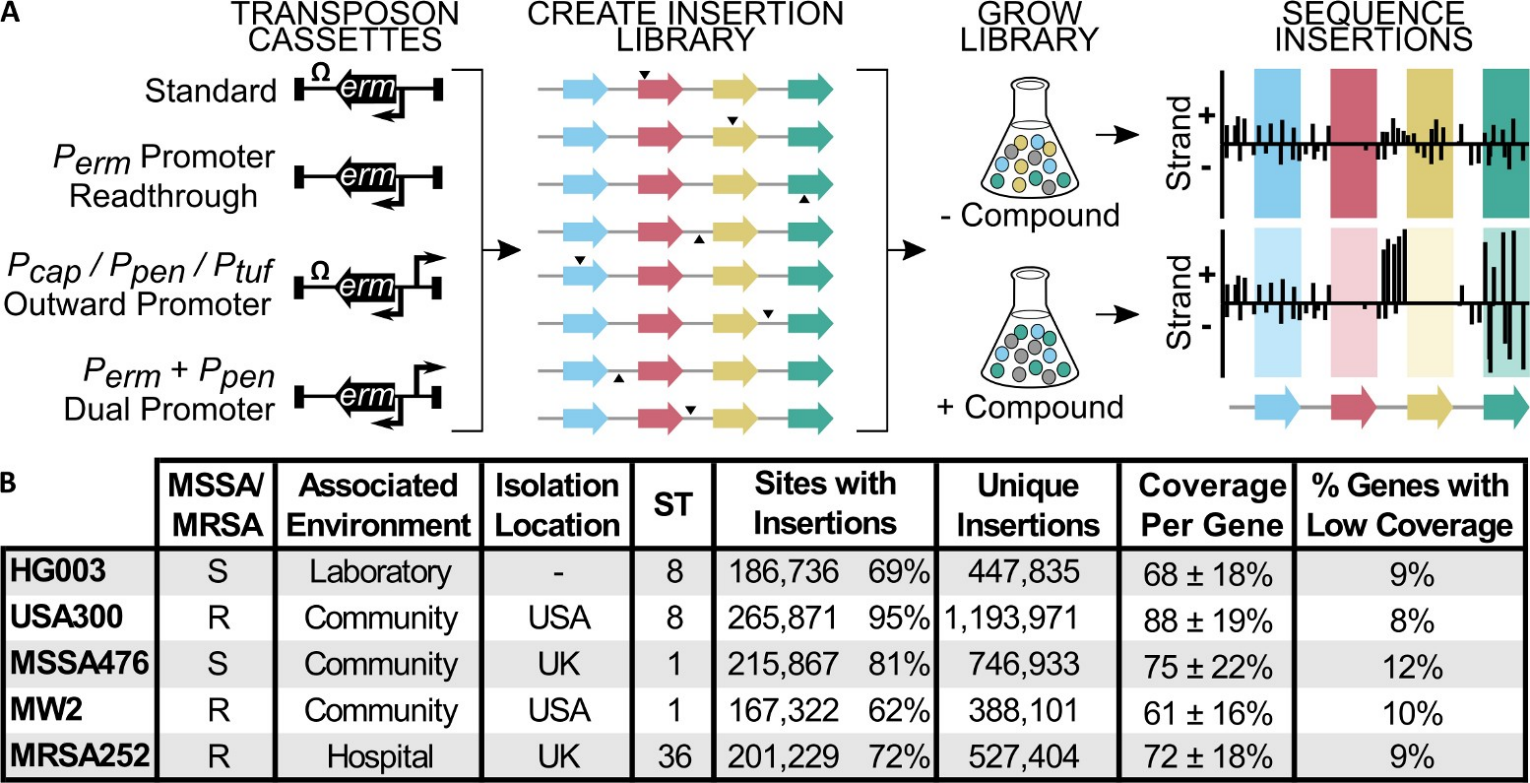
We created complex transposon libraries of similar coverage in five diverse *S. aureus* strains. (A) Six barcoded transposon constructs were used to make sub-libraries that were combined into a single transposon library. Construct 1 can drive expression only of *erm^R^* (gene within the transposon constructs). Constructs 2–6 can drive expression of other genes, either by readthrough of *erm^R^* in constructs lacking terminators or because they contain an additional outward-facing promoter (*P_cap_*, *P_pen_*, or *P_tuf_*). The libraries can be sequenced to find essential genes that lack transposon insertions (red gene) or treated with compound and then sequenced to find genes that sustain growth in the condition (yellow gene), upregulation signatures (upstream of yellow gene), and genes with conditional fitness costs (green gene). (B) High-density transposon libraries were made in five *S. aureus* strains. MSSA = methicillin-sensitive *S. aureus*; MRSA = methicillin-resistant *S. aureus*; ST = sequence type. Sites with insertions refers to the number of TA dinucleotide sites having insertions of at least one transposon construct. The “unique insertions” column sums the TA insertion counts from each transposon construct. Coverage per gene is the average percent of TA sites within a gene with insertions, ± standard deviation. Low-coverage genes were defined as those that had less than half of the average gene coverage.

Here we have used our previously reported platform for creating transposon libraries in *S*. *aureus* to make high-density transposon libraries in five strains [17]. These libraries comprise six sub-libraries, each made using a different transposon construct. Several constructs contain outward-facing promoters, which minimize polar effects within polycistronic operons. In addition, under some conditions, insertions of outward-facing promoter constructs can upregulate nearby genes to provide a distinct fitness advantage that is mechanistically informative [21]. The five *S*. *aureus* strains we chose represent three clonal complexes (Fig 1B), including two sets of MRSA/methicillin-sensitive (MSSA) strains from the same sequence type. MW2, from sequence type 1 (ST1), was the first lineage of community-acquired MRSA infections reported in the United States [22–24]. MSSA476, a representative of a United Kingdom community-acquired MSSA lineage, is likewise from ST1 [25]. We have chosen USA300-TCH1516 to represent the hypervirulent USA300 lineage from ST8, which has become the most common community-acquired MRSA lineage in the United States [23, 26, 27]. The commonly used MSSA lab strain HG003 is also ST8. To capture a larger scope of the *S*. *aureus* phylogeny, we also included MRSA252, an ST36 hospital-acquired MRSA strain predominantly found in Europe that has attracted attention for its unusually large accessory genome (S1 Fig) [25].

We found 200 genes that scored as essential in all five strains. We also identified genes that were uniquely essential in a given strain, as well as genes and pathways present in all five strains but essential in a subset. These cases imply that strain-specific distal genetic determinants, whether allelic in nature or horizontally acquired as with β-lactam resistance cassettes, can reshape the role of even core pathways. The lipoteichoic acid (LTA) pathway is one example of a conserved pathway that is differentially important across *S*. *aureus* strains.

We probed to what extent the variable genetic dependencies evident in the essential gene analysis shaped mutant fitness upon exposure to daptomycin, an antibiotic used for *S*. *aureus* infections. Patients who cannot tolerate β-lactam or vancomycin treatment, or who have resistant infections, are commonly treated with daptomycin. Although still rare, daptomycin nonsusceptibility is on the rise [6]. It might be possible to overcome infections that are resistant to daptomycin by co-administering a compound that targets an intrinsic resistance factor found to be universally important in withstanding daptomycin stress, particularly if that factor is itself important for pathogenesis. A recent study comparing two *Streptococcus pneumoniae* strains found major differences in factors that influenced daptomycin susceptibility, raising questions about the prevalence of universally conserved daptomycin intrinsic resistance factors in *S*. *aureus* [28]. We identified daptomycin intrinsic resistance factors conserved in all strains and demonstrated that even pathways that appear variably essential among strains under normal growth conditions can become crucial in all upon exposure to daptomycin, illuminating a path forward for combatting daptomycin resistance in *S*. *aureus*.

## Results

### Creation of high-density *S*. *aureus* transposon libraries with both positive and negative modulation of gene expression

To characterize the functional diversity of *S*. *aureus*, we generated transposon libraries with similar coverage in five strains (HG003, USA300-TCH1516, MSSA476, MW2, and MRSA252). For each strain, we used our phage-based transposition method to make six sub-libraries using different transposon constructs (Fig 1A) [17]. One transposon construct contained an erythromycin resistance gene driven by its own promoter (*P*_*erm*_) and followed by a transcriptional terminator; another lacked the transcriptional terminator to allow readthrough from the *P*_*erm*_ promoter; three constructs contained the transcriptional terminator, but included an additional, outward-facing promoter that varied in strength; and the sixth construct lacked the transcriptional terminator and had an added outward-facing promoter, allowing for bidirectional transcription. The sub-libraries were made separately and then pooled, with unique barcodes in each transposon construct to enable disambiguation. These high-density composite libraries often contained transposon insertions from multiple constructs at a given TA insertion site. The constructs containing outward-facing promoters minimized polar effects by allowing expression of downstream genes when an operon was disrupted. Under some conditions, they also provided supplementary fitness information. For example, under antibiotic exposure, an upregulated gene conferring a growth advantage presented in the data as a strand-biased enrichment of promoter-containing transposon insertions upstream of the gene. These “upregulation signatures” can identify molecular targets or mechanisms of resistance (Fig 1A, yellow gene; Methods).

Tn-Seq analysis showed that pooling the six transposon sub-libraries afforded libraries with high coverage in every genome (Fig 1B). Although there was a three-fold difference in unique insertions, counting insertions from each sub-library as distinct, between the library with the fewest insertions (MW2) and the library with the most insertions (USA300-TCH1516), approximately 90% of the genes in each genome had high coverage of TA dinucleotide insertion sites. This gave us confidence that we could obtain a reliable estimate of gene essentiality differences across the strains.

### Essential gene analysis reveals unexpected differences between strains

**Fig 2:**
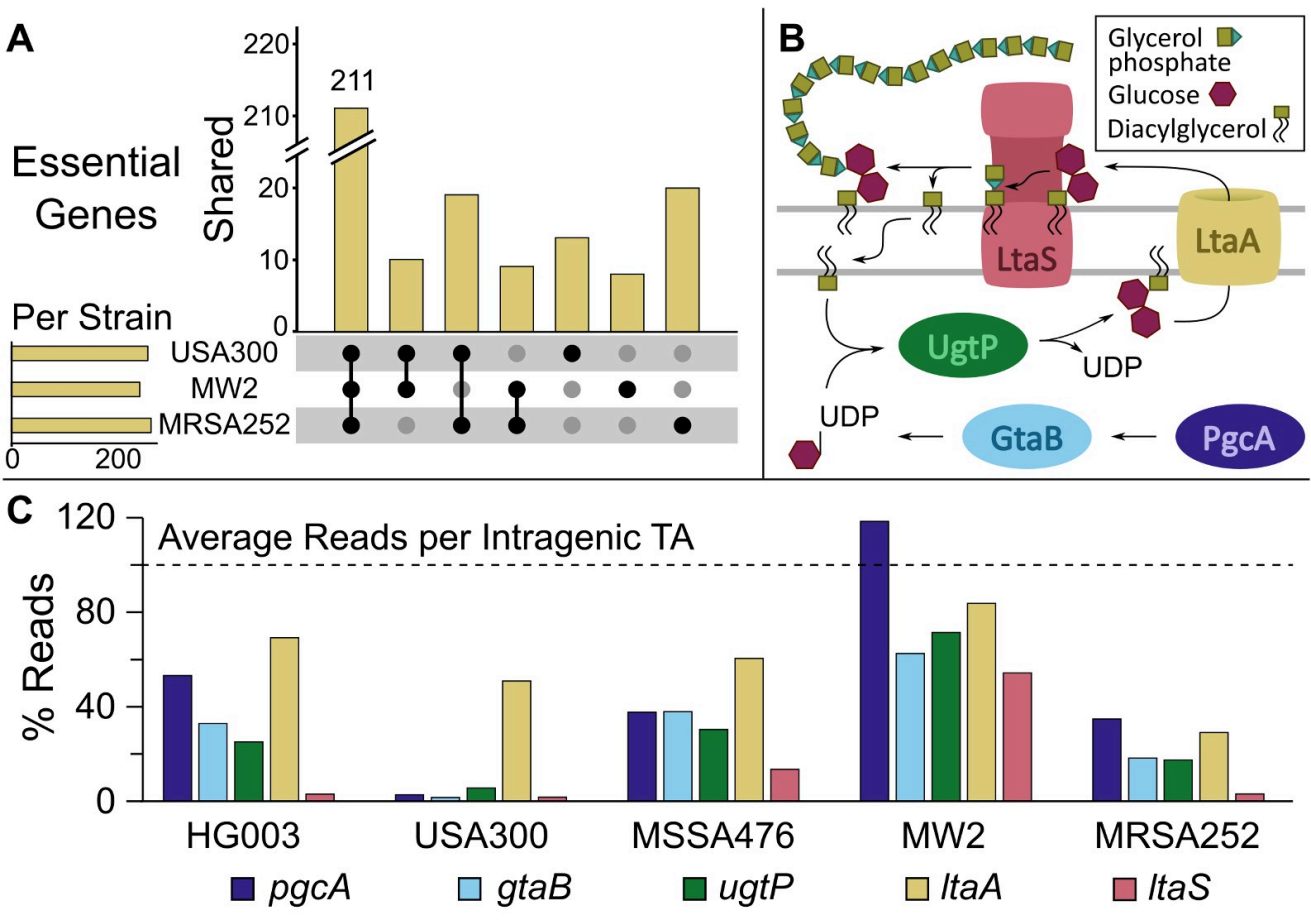
The fitness of lipoteichoic acid pathway mutants varies by strain. (A) The number of essential genes per strain (horizontal bars) and the number of genes found to be essential for all three, two, or one of the MRSA strains (vertical bars, with black dots denoting strains for which the genes are essential). Only MRSA strains were included for simplicity, but a similar plot with the MSSA strains is included in the supporting information. Note that gene essentiality is functionally defined as a lack of transposon insertions in a gene, but it may be possible to make knockouts of some of these genes under favorable conditions. (B) A schematic of the lipoteichoic acid pathway in *S. aureus*. (C) The number of reads in each LTA pathway gene for each transposon library, expressed as a percentage of the average number of reads per TA site in the coding regions of the libraries. There are strain-dependent differences in transposon insertions in LTA pathway genes, with USA300-TCH1516 appearing reliant on most genes in the pathway while MW2 is insensitive even to inactivation of *ltaS*.

We performed Tn-Seq analysis on the five transposon libraries to identify essential genes in each strain. For the purposes of this study, we defined an essential gene as one whose loss precluded survival or multiplication in competition with a heterogeneous bacterial population. We categorized genes as essential, non-essential, or indeterminate based on TA insertion site coverage and sequencing reads (S1 Table). 200 genes were categorized as essential under aerobic growth conditions in all five strains (S2 Fig, S2 Table); an additional 11 genes were required for at least the three MRSA strains (Fig 2A). Insofar as it is possible to define by Tn-Seq, the 200 genes found to be essential in all five strains represent the core essential genome of *S*. *aureus*. However, as in any essential gene list from genome-wide mutagenesis, there are both false positives and false negatives in this core essential genome. A known false positive is *tarO*, which encodes the first gene in the wall teichoic acid biosynthesis pathway. It is possible to delete *tarO in vitro*, but mutants have substantial growth defects [29] that evidently preclude survival in a competitive Tn-Seq experiment. A known false negative is *murG*, which is required to make the peptidoglycan precursor Lipid II [30]. This gene was found to be essential in four strains and indeterminate in one, leading to its inclusion with 38 other genes which were categorized as essential or uncertain (indeterminate) depending on the strain (S2 Table). Because there are fewer possible insertion sites, short genes are more often categorized as uncertain. This may explain the presence of *rps* genes encoding ribosome components in the list of 39. Essential genes that are relatively long can also be categorized as indeterminate due to inevitable read mapping errors that result in apparent insertions in them. In any event, appropriate use of Tn-Seq essential gene lists requires understanding that they are based on apparent viability of mutants as judged by transposon reads in experiments where millions of reads are mapped at a time.

Gene ontology overrepresentation analysis showed that the genes found to be essential in all strains were enriched in DNA, RNA, and protein metabolic processes (S3 Table). Given their roles in central metabolic pathways, many of these genes are likely to be essential under all growth conditions. Some, however, such as those involved in aerobic respiration, may only be essential under certain conditions. Phospholipid and monosaccharide synthesis were also enriched pathways, reflecting the importance of fatty acids and other cell envelope precursors. Hypothetical genes were significantly underrepresented among the ubiquitously essential genes but dominated the group of essential genes present in a subset of the genomes.

Among annotated genes present in all five strains, there were notable differences in their importance. We used a statistical test to verify apparent differences in gene essentiality between strains (Methods). Strains of the same sequence type displayed as much variability in gene essentiality as strains from different sequence types (S2 Fig), suggesting it may not be possible to draw general conclusions about a clonal complex from a functional genomics analysis of one member. Variably essential genes were found in multiple pathways. For example, the uniquely essential genes in MRSA252 included *sbcD*, which is homologous to an *E*. *coli* gene encoding a DNA hairpin cleavage enzyme [31, 32]; *sagB*, which encodes a β-N-acetylglucosaminidase [33]; and *gdpP*, which hydrolyzes the bacterial second messenger cyclic-di-AMP [34]. While an essentiality designation in a Tn-Seq analysis does not necessarily imply that individual knockouts are not viable (*e*.*g*., *tarO* mutants), it does suggest substantial differences in mutant fitness across strains.

**Fig 3:**
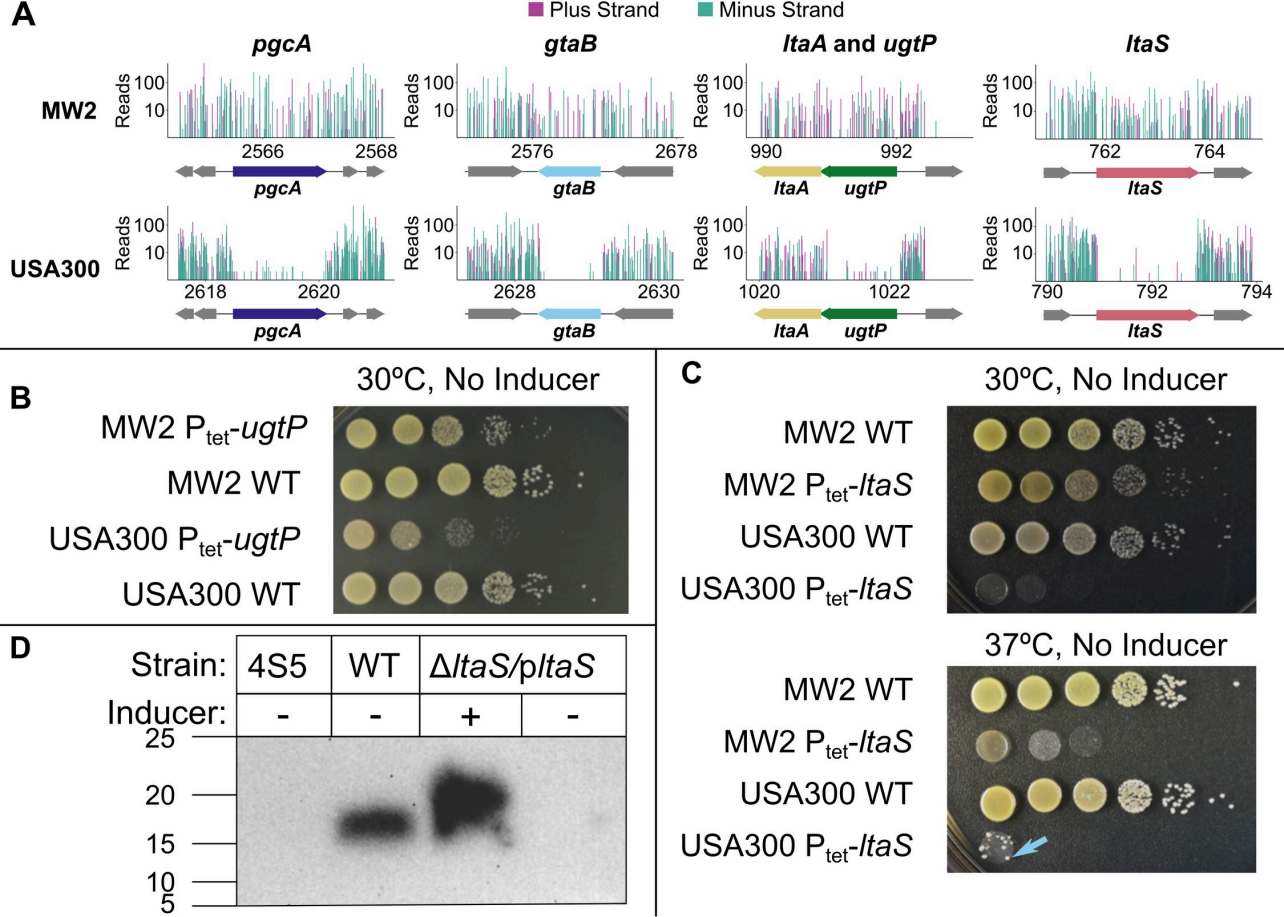
The lipoteichoic acid pathway is crucial for fitness of USA300-TCH1516 but is dispensable in MW2. (A) Tn-Seq data for the LTA pathway genes, with sequencing reads from insertions in the plus strand in purple and minus strand in teal. The reads were calculated by summing two replicate Tn-Seq experiments, only including data from transposon constructs that contained a transcriptional terminator. The data were normalized to each other by non-zero means normalization prior to plotting. The x-axis is expressed in kilobases. The y-axis is expressed on a log_10_ scale and is truncated to 500 reads. (B) Growth on agar plates for wild-type (WT) and Δ*ugtP* strains confirms that USA300-TCH1516 is more sensitive to *ugtP* deletion than MW2 is. (C) Growth on agar plates of wild-type and Δ*ltaS* strains confirms that *ltaS* is dispensable in MW2 at both 30°C and 37°C. Arrow highlights a suppressor mutant growing in the USA300 spot. (D) Western blot of LTAs produced by 4S5 (known to not produce LTAs), wild-type MW2, and MW2 P_tet_-*ltaS* confirms that MW2 P_tet_-*ltaS* does not produce LTAs unless expression of the exogenous copy of *ltaS* is induced.

One pathway found to be differentially important by Tn-Seq was the LTA biosynthesis pathway. LTAs are long glycerol or ribitol phosphate polymers anchored in the membrane of Gram-positive bacteria. In *S*. *aureus*, LTAs are important in cell division, autolysin regulation, and virulence, among other processes [35, 36]. The LTA pathway consists of five genes: *pgcA*, *gtaB*, *ugtP (ypfP)*, *ltaA*, and *ltaS* (Fig 2B). The first three genes encode proteins involved in the biosynthesis of diglucosyl-diacylglycerol (Glc_2_DAG), the membrane anchor on which LTAs are assembled. Glc_2_DAG is made inside the cell and flipped to the cell surface by LtaA. The LTA synthase, LtaS, then assembles the polymer by sequential transfer of phosphoglycerol units from phosphatidylglycerol to Glc_2_DAG. LTAs can then be further modified on the extracellular surface by D-alanine substitutions and N-acetyl glucosamine residues [37, 38]. If Glc_2_DAG is not available due to deletion of an upstream gene, LtaS can synthesize LTAs on the alternative membrane anchor phosphatidylglycerol. The four genes upstream of *ltaS* were previously found to be nonessential for *S*. *aureus* viability; however, strains lacking the Glc_2_DAG anchor normally present in LTA were shown to have substantial pathogenesis defects [39–42]. Previous findings about the importance of *ltaS* are less clear. One study reported that *ltaS* can be deleted at 30°C; however, other studies have found that even at 30°C osmotically-stabilizing conditions are required for growth of *ltaS* mutants [43–46]. Our Tn-Seq analysis showed that mutant fitness within the LTA pathway varied considerably by strain (Fig 2C). The *ltaA* gene was expendable in all strains, but the three genes responsible for synthesis of the Glc_2_DAG anchor, *pgcA*, *gtaB*, and *ugtP*, were classified as essential only in USA300-TCH1516 (Fig 3A, S3 Fig), a pattern suggesting a greater reliance on intracellular Glc_2_DAG in this strain. Additionally, even though the library was grown at 30°C, which one report identified as a permissive temperature for growth of *ltaS* knockouts [43], we found that *ltaS* was only dispensable in two strains, MW2 and MSSA476 (Fig 3A, S3 Fig).

To confirm fitness differences between strains, we tested viability of deletion mutants in the LTA pathway. Consistent with results in other USA300 strain backgrounds, we were able to delete *ugtP* from USA300-TCH1516, but a spot dilution assay showed that the mutant was less fit than the corresponding mutant in MW2 (Fig 3B). We also confirmed that *ltaS* is dispensable in MW2, but essential in two other tested strains at 30°C (Fig 3C, S4 Fig). Although fitness decreased substantially with increased temperature, the MW2Δ*ltaS* strain also grew at 37°C (Fig 3C). We did not find any evidence for a duplication of *ltaS* in MW2 and confirmed that the MW2Δ*ltaS* mutant does not produce LTAs (Fig 3D). Because lethality due to *ltaS* deletion can be suppressed by deletion of *gdpP*, *clpX*, *mazE*, or *sgtB* [44–46], we examined the sequences of these genes in the five strains. There were no differences between MW2 and the other strains in *gdpP*, *mazE*, or *sgtB;* however, for *clpX* we found that both MW2 and MSSA476 had a nonsynonymous SNP compared with the other three strains, resulting in a change of aspartate for asparagine at position 339 in the protein. ClpX was reported to be important for fitness of wild-type *S*. *aureus* at 30°C [45], and it seemed unlikely that this mutation inactivated ClpX to suppress *ltaS* deletion. However, we introduced the *clpX* allele found in HG003 into MW2Δ*ltaS* and assessed fitness using a spot dilution assay. When the HG003 *clpX* allele was expressed, MW2Δ*ltaS* fitness decreased at 30°C and 37°C (S5 Fig). We concluded that the difference in *clpX* sequence may contribute to the dispensability of *ltaS* in MW2. Additional studies will be required to identify the key factors that make *ltaS* less important in some strains than others.

### *S*. *aureus* strains share key daptomycin resistance factors

Compounds that sensitize bacteria to existing antibiotics may be useful as components of therapeutic regimens even when they do not have antibiotic activity on their own [47, 48]. We wondered whether it would be possible to identify targets for compounds that increase daptomycin susceptibility by growing the library in its presence. Daptomycin is a calcium-dependent lipopeptide antibiotic frequently used to treat *S*. *aureus* infections. Although its mechanism of action is not completely clear, it has been shown to insert into Gram-positive bacterial cell membranes and change membrane curvature, mislocalize cell division and cell wall synthesis proteins, depolarize the membrane, and ultimately kill the cell [49–54]. The majority of *S*. *aureus* strains remain susceptible to daptomycin, but there have been steady reports of daptomycin nonsusceptibility [55].

**Fig 4:**
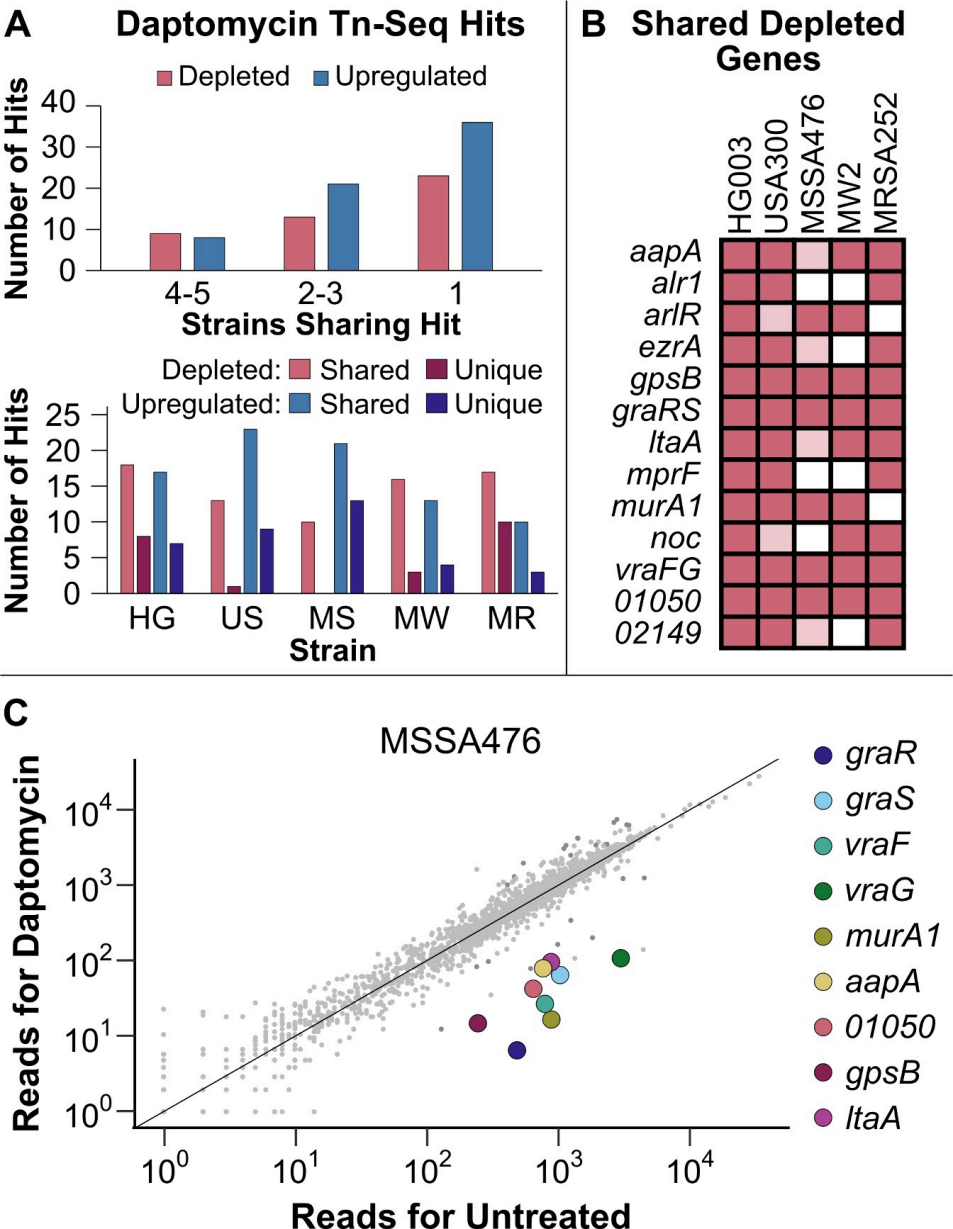
Transposon sequencing supports previously reported vulnerabilities in *S. aureus* and reveals new ones. (A) A comparison of the Tn-Seq hits under daptomycin exposure in five strains of *S. aureus*. All hits needed to have a q-value less than 0.05. Depleted genes had 10-fold fewer reads in the daptomycin-exposed sample compared to the control sample after normalization. Upregulated genes had a strand-biased enrichment of reads in the region upstream of the gene. The upper graph shows how many strains each hit was found in while the lower graph shows how many hits found in each strain were shared with at least one other strain and how many were unique to that strain. (B) Genes that were depleted of reads under daptomycin exposure in at least three of the transposon libraries, using the cutoffs described above. Hypothetical genes are listed according to their NCTC 8325 locus tag numbers. Genes meeting all cutoffs in a strain are indicated by dark pink. Those that have a significant q-value but are only depleted 5- to 10-fold are indicated in light pink. (C) Normalized Tn-Seq reads from daptomycin-exposed MSSA476 in RPMI+LB plotted against the reads from the control condition. Genes from above that were shared by at least 4 strains are highlighted.

We grew the five *S*. *aureus* transposon libraries in the presence and absence of sub-minimum inhibitory concentration (MIC) daptomycin and in two different media at 37°C (Methods). We found substantial overlap in the significantly depleted and upregulated genes in the two media, so the results were considered as a union of the two conditions. For every strain, there were more hits shared with at least one other strain than there were hits unique to that strain (Fig 4A, S4 and S5 Tables). Genes that were substantially depleted (>10-fold) in three or more strains are shown in Fig 4B. Several vulnerabilities were above or very close to the 10-fold cutoff in all five strains.

**Fig 5:**
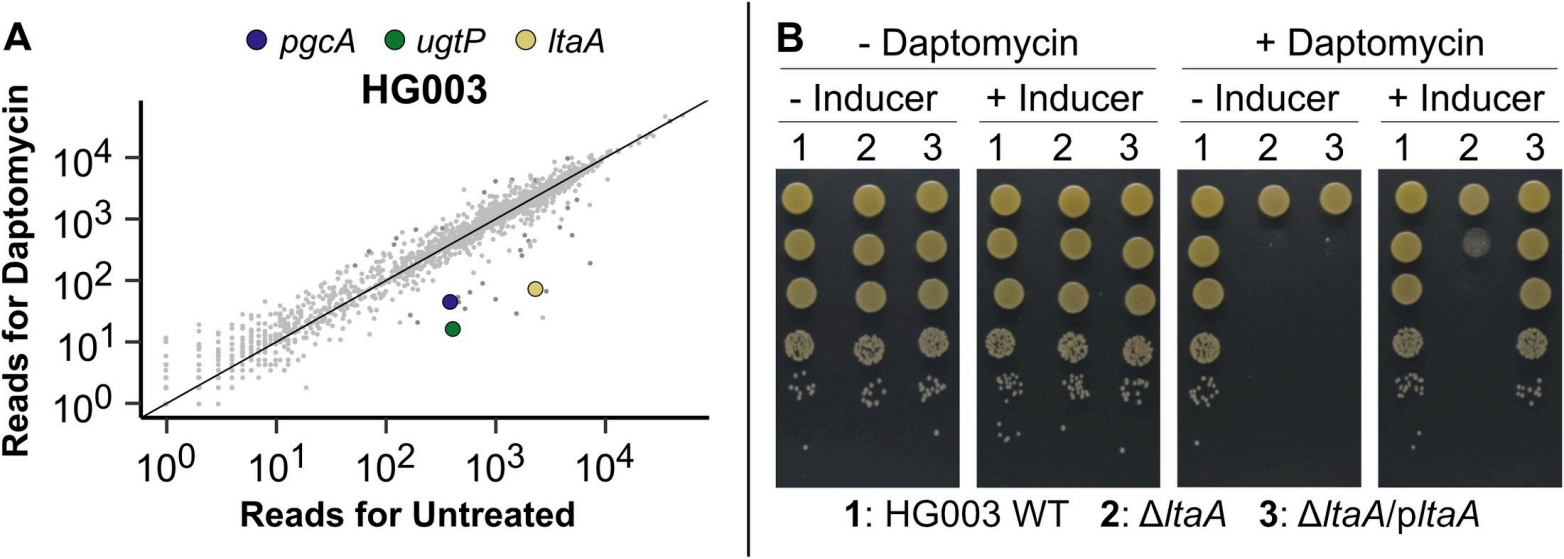
LTA loss sensitizes *S. aureus* to daptomycin. (A) Normalized Tn-Seq reads from daptomycin-exposed HG003 in RPMI+LB plotted against the reads from untreated HG003 in the same growth medium. Genes in the LTA pathway that were significantly depleted are highlighted. (B) Growth on agar plates at 37°C of HG003 wild-type (WT), Δ*ltaA*, and inducible *ltaA* complementation strains in the presence or absence of 2.5 μg/mL daptomycin and the inducer confirms that *ltaA* is required for *S. aureus* growth under daptomycin exposure.

Many of the shared vulnerabilities to daptomycin are in genes related to the cell envelope. These include *graRS/vraFG*, encoding the multi-component signaling system that regulates cell envelope processes [56–58]; *arlR*, encoding a regulator of virulence genes [59, 60]*; murA1*, encoding an enzyme that catalyzes the first step in peptidoglycan biosynthesis [61, 62]; *alr1*, one of two genes encoding an alanine racemase that supplies D-alanine for cell wall synthesis [63]; *mprF*, encoding a phosphatidylglycerol lysyltransferase that modifies membrane charge [64]; and *ltaA*, encoding the Glc_2_DAG flippase gene in the LTA pathway [39]. The earlier LTA pathway genes, *gtaB*, *pgcA*, and *ugtP*, were also significantly depleted in MW2, with the latter two also depleted in HG003 (Fig 5A). In addition, we identified three depleted cell division regulators, *gpsB*, *ezrA*, and *noc* (Fig 4B and 4C) [65–67]. A number of these depleted genes have been previously associated with daptomycin susceptibility, including *mprF* [68, 69], *graRS*/*vraFG* [57, 70], and *ezrA* [71], but some that were substantially depleted in all five strains have not been connected to daptomycin, including *gpsB* and *ltaA*. Using spot dilution assays, we tested mutants in several genes including *ezrA*, *gpsB*, *graR*, *ltaA*, *mprF*, *vraFG*, *SAOUHSC_01050*, and *SAOUHSC_02149* against daptomycin and confirmed increased susceptibility (S6 Fig).

We examined *ltaA* in more detail to assess the degree to which it increased daptomycin susceptibility. Tn-Seq is highly sensitive to fitness differences of mutants and, because spot dilution assays are also highly sensitive, they are a good way to confirm fitness differences. However, an apparently large difference in mutant fitness under antibiotic treatment may translate to a minor effect on MIC. We first confirmed increased susceptibility of *ltaA* mutants in HG003, MW2, and USA300-TCH1516 to daptomycin using spot dilution assays and showed that increased sensitivity can be reversed by *ltaA* complementation (Fig 5B, S7 Fig). In liquid medium, *ltaA* mutants in HG003 and MW2 had the same daptomycin MIC as wild type, but showed a several hour delay in entry into the exponential phase of growth compared with wild type (S8 Fig), a finding consistent with the greatly reduced growth of the *ltaA* mutants in the transposon libraries. The explanation for the lag in growth of *ltaA* mutants requires further investigation to assess whether it will be possible to target the LTA pathway to overcome daptomycin nonsusceptibility, but it is clear that Glc_2_DAG-LTA in the cell membrane contributes to *S*. *aureus* survival in the presence of daptomycin.

Just as there were more shared than unique depleted genes for each strain, there were more shared than unique upregulated genes (Fig 4A). The outward-facing promoters in several of our transposon constructs can upregulate downstream genes, allowing us to identify genes that confer a fitness advantage when upregulated. They are detected by strand-biased read enrichments in promoter-containing constructs, and we have previously confirmed that these strand-biased read enrichments, or upregulation signatures, do reflect increased expression of proximal genes [21]. Often the genes identified this way are the targets of the antibiotic itself or reveal gain-of-function resistance mechanisms. Standard transposon libraries cannot identify gain-of-function resistance mechanisms, although loss-of-function resistance mechanisms can be identified through intragenic read enrichments. Here we found that the genes enriched in reads upon daptomycin exposure belonged to a wide assortment of cellular processes and we could not discern a pattern amongst them (S6 Table).

Many of the genes for which we identified upregulation signatures in multiple strains were previously implicated in daptomycin resistance in *S*. *aureus* (Table 1). Daptomycin nonsusceptibility normally develops through the acquisition of multiple independent mutations that result in changes to the cell envelope [55], and many of the mutations are thought to be gain-of-function mutations that result in increased activity. For example, mutations in *mprF* have been selected *in vitro* and in the clinic and are thought to increase lysyl phosphatidylglycerol in the outer leaflet of the membrane [72]. Other gain-of-function resistance mechanisms identified in our Tn-Seq libraries include *cls2*, one of two cardiolipin synthases in *S*. *aureus* [73]; the staphyloxanthin biosynthesis gene *crtM* [74]; and two signaling systems, *graRS* and *walKR* (*yycFG*) [70, 75]. Some of these resistance mechanisms have been reported for daptomycin resistance in other bacteria as well [76]. These hits affirm the utility of upregulation signatures for identifying clinically-relevant mechanisms of resistance. The gene *murA2*, whose function was identified as important for withstanding daptomycin exposure in the depletion analysis, also had upregulation signatures in several strains. The importance of MurA activity identified in this study through both depletion and upregulation analysis is consistent with recent studies showing that a combination of daptomycin and fosfomycin is significantly more effective at curing MRSA bacteremia than daptomycin alone, leading to an ongoing phase III clinical trial [77–79]. One caveat of the upregulating transposons is that they may have distal effects, upregulating both the most proximal gene and other downstream genes, especially within operons. Individual mutants need to be tested to confirm that the proximal gene is most responsible for the phenotype. For example, we identified an upregulation signature for *ugtP* in USA300-TCH1516 and in MSSA476. The *ugtP* gene is upstream of *ltaA* in an operon and it is unclear whether upregulating *ugtP* on its own would recapitulate the transposon-induced fitness advantage or whether *ltaA* upregulation is also necessary. Regardless of whether upregulation of one or both genes in the operon drives the fitness advantage, the result is consistent with findings from the depletion analysis that Glc_2_DAG-LTA is important for withstanding daptomycin stress.

**Table 1:** Upregulation signatures identify genes previously linked to reduced susceptibility to daptomycin.

Gene	Function	HG003	USA300	MSSA476	MW2	MRSA252
*cls2*	Cardolipin synthesis					
*crtM*	Staphyloxanthin synthesis					
*graRS*	Cell wall stress response					
*mprF*	Lysyl phosphatidylglycerol synthesis					
*murA1*	Rate-limiting step in peptidoglycan precursor synthesis					
*murA2*						
*walKR*	Cell wall stress response					
*00969*	Unknown					
*02149*	Unknown					

Only showing genes with upregulation signatures in at least two strains. Strains sharing upregulation signature indicated with blue shading. Upregulation and/or suspected gain-of-function SNPs in all of these genes were previously found to confer a survival advantage in the presence of daptomycin. Hypothetical genes are listed according to their NCTC 8325 locus tag numbers.

Upregulation signatures were found upstream of the genes *SAOUHSC_02149* and *SAOUHSC_00969* in all five strains. These genes each encode a small protein of unknown function with a single transmembrane helix. We previously identified both as daptomycin resistance factors in a Tn-Seq study of a single *S*. *aureus* strain and confirmed that upregulation confers resistance [21]. The observation that these genes are important for daptomycin resistance in all five *S*. *aureus* strains suggests some effort should be devoted to evaluating the physiological roles of their encoded products.

## Discussion

Declining sequencing costs and efficient transposon mutagenesis methodologies enable investigation of the genetic basis of phenotypes on a large scale. Tn-Seq experiments are typically conducted in a single strain that is considered representative, and it is assumed that results from that strain can be extrapolated to other members of the species. Recent studies have challenged this notion, demonstrating clear strain-dependent gene requirements in *Mycobacterium tuberculosis*, *Streptococcus pneumoniae*, and *Pseudomonas aeruginosa* [19, 20, 28].

In the present study, we identified a number of strain-dependent differences in apparent essentiality of cell envelope and other genes. Due to our general interest in the bacterial cell envelope, we focused on the LTA pathway. Although *S*. *aureus* can survive with low levels of LTA [36], and one study reported that a laboratory strain can grow at 30°C after *ltaS* deletion [43], other studies have found LTA to be required for growth in the absence of suppressor mutations [44–46]. We observed large differences in *ltaS* essentiality across strains even at 30°C, with MW2 being notable for containing a large number of insertions while three other strains contained almost none. Whether strains that can survive without LTA *in vitro*–either because they contain known suppressors or have other, uncharacterized genetic background differences that make them viable–can survive in the more challenging environment of an animal infection needs to be assessed.

We have suggested that targeting upstream steps in the LTA pathway may be beneficial in combination with daptomycin. Across all strains, daptomycin treatment differentially reduced growth of transposon mutants in genes upstream of *ltaS*, and we confirmed that daptomycin adversely impacts growth of strains that lack the Glc_2_DAG anchor in LTA. Moreover, other studies have shown that *S*. *aureus* that lacks the Glc_2_DAG anchor of LTA have attenuated pathogenicity [39, 42], providing another reason to consider that targeting upstream genes in the LTA pathway could be useful. How evolution of daptomycin non-susceptibility is affected by mutations in this pathway remains to be investigated.

We have also demonstrated an improved method for identifying gain-of-function resistance mechanisms via Tn-Seq. The upregulation signatures that we found under daptomycin exposure using our transposons with outward-facing promoters largely echoed the depletion hits. That is, if removing a gene sensitizes a bacterium to an antibiotic, upregulating that gene is often protective. For daptomycin, intrinsic resistance factors are predominantly cell envelope, cell division, and stress response system genes. Although a standard Tn-Seq library can identify intrinsic resistance factors through sensitization of knockouts, sensitization readouts can only look at genes where a decline in insertions can be detected. Upregulation signatures, on the other hand, are indifferent to gene essentiality. For example, *ugtP* was upregulated under daptomycin stress in USA300-TCH1516 even though it contained few insertions and so was categorized as essential in that strain. Moreover, *walR*, an essential multicomponent signaling system gene, was upregulated in multiple strains. As validation for the utility of upregulation signatures, most of the genes that had upregulation signatures are previously known or suspected gain-of-function resistance mechanisms for daptomycin.

In summary, we note that the comparative data provided here represents the most extensive genome-wide functional analysis of *S*. *aureus* gene dependencies across strains. Other studies have compared multiple genomes, but without the benefit of information on the relative importance of different pathways. We predict that Tn-Seq results across multiple strains will complement findings from other systems biology approaches [9, 80]. As the cost of sequencing continues to decrease, performing Tn-Seq in multiple strains will become routine and we expect that this will lead to a more comprehensive understanding of the scope of bacterial phenotypic diversity. We hope the information will lead to improvements in development and use of compound combinations for antibiotic therapy, which we expect will become the norm to counteract resistance.

## Materials and methods

### Materials, bacterial strains, plasmids, and oligonucleotides

All reagents were purchased from Sigma-Aldrich unless otherwise indicated. Bacterial culture media were purchased from BD Sciences. Restriction enzymes and enzymes for Tn-Seq preparation were purchased from New England Biolabs. Oligonucleotides and primers were purchased from Integrated DNA Technologies. DNA concentrations were measured using a NanoDrop One Microvolume UV-Vis Spectrophotometer (Thermo Scientific). DNA sequencing was performed by Eton Bioscience unless otherwise noted. KOD Hot Start DNA polymerase (Novagen) was used for PCR amplification. *E*. *coli* strains were grown with shaking at 37°C in lysogeny broth (LB) or on LB plates. *S*. *aureus* strains were grown at 30°C in tryptic soy broth (TSB) shaking or on TSB agar plates unless otherwise noted. For *S*. *aureus* strains, compounds for selection or gene induction were used at the following concentrations: 5 μg/mL chloramphenicol and erythromycin; 50 μg/mL kanamycin and neomycin; or 2.5 μg/mL tetracycline. For *E*. *coli* strains, 100 μg/mL carbenicillin was used for selection. Daptomycin was purchased from Sigma-Aldrich and Calbiochem. The bacterial strains, plasmids, and oligonucleotide primers used in this study are summarized in S7 Table.

### Transposon library construction

Construction of the transposon library in the laboratory *S*. *aureus* strain HG003 has been described [17]. A similar strategy was used to make libraries in other *S*. *aureus* genetic backgrounds after deleting non-compatible DNA restriction systems and endogenous antibiotic resistance genes that would interfere with transposon library construction. In the community-acquired *S*. *aureus* MW2 and MSSA476 strains, the clonal complex CC1 *hsdR* Type I restriction system in each background was first deleted using the temperature sensitive shuttle vector pKFC with ~1 kb DNA homology flanking arms. A second restriction system unique to MSSA476 was deleted in a subsequent round using pKFC to generate the transposon library host. In both cases, pKFC was passaged through the laboratory strain RN4220. Libraries were constructed in the *S*. *aureus* community-acquired strain USA300-TCH1516 by first curing the endogenous plasmid pUSA300HOUMR through destabilizing replication via integration of pTM283. Cointegrated pUSA300HOUMR-pTM283 was passaged at 30°C for two rounds of outgrowth in 10 mL of TSB media before streaking to single colonies. Colonies exhibiting kanamycin and erythromycin sensitivity (encoded on pUSA300HOUMR) were further checked by PCR to confirm loss of plasmid. The resulting strain, TM283, was used as host for transposon library construction. A transposon library of the hospital-acquired, methicillin-resistant *S*. *aureus* MSRA252 strain was made by first converting the *hsdR* (SAR0196) Type I restriction gene deletion shuttle vector pGKM305 into a high frequency transduction vector by adding a φ11 DNA homology region (primers GKM422-423) into the SfoI site to make pGKM306. The plasmid was electroporated into RN4220 and transduced into wild-type MRSA252 using φ11-FRT with temperature permissive selection at 30°C [17]. This temperature was chosen to minimize false positives that could occur by conducting the process at a higher temperature. The SAR0196 gene was then deleted as described above. Both copies of the endogenous duplicated erythromycin resistance gene (SAR0050 and SAR1735) were likewise deleted in two rounds using pKFC_SAR0050, except plasmids were directly electroporated into the restriction negative parent strain GKM361. Due to endogenous aminoglycoside resistance, the transposase expressing plasmid pORF5 Tnp+ was converted into chloramphenicol resistant plasmids to enable selection in strain TXM369 and library construction.

### Transposon sequencing

To identify the essential genes in each of the *S*. *aureus* strains (HG003, USA300-TCH1516, MSSA476, MW2, and MRSA252), library aliquots were thawed and diluted to an OD_600_ between 0.2 and 0.3 in 10 mL of cation-adjusted Mueller Hinton broth (MHBII) in duplicate. MHBII was chosen as it is the standard medium for determining MIC for antibiotics in clinical laboratories. The cultures were then incubated shaking at 30°C until they reached an OD_600_ of approximately 0.4, roughly 1.5–1.75 hours. This temperature was chosen to produce a conservative list of essential genes, as many mutants survive better at 30°C than at higher temperatures. The cells were pelleted, and the DNA was extracted and prepared for Tn-Seq as previously described [17]. Samples were then submitted to either the Harvard Biopolymers Facility or the Tufts University Core Facility for sequencing on a HiSeq 2500 instrument.

To identify genes affected by daptomycin exposure, transposon library aliquots for the six strains were inoculated in either MHBII or Roswell Park Memorial Institute media supplemented with 10% LB (RPMI+LB). The cultures were incubated for 1.5 hours at 30°C in a shaking incubator as described above. The cultures were diluted to OD_600_ 0.005 in either 2 mL MHBII or RPMI+LB supplemented with 0.5 mM CaCl_2_ and 0.5 mM MgCl_2_ and containing a series of daptomycin concentrations (μg/mL): 0, 0.12, 0.25, 0.5, and 1. The cultures were then incubated at 37°C in a shaking incubator and their ODs were monitored using a Genesys 20 spectrophotometer (Thermo Scientific). The daptomycin MIC for all five strains was approximately 1–2.5 μg/mL under the conditions used in Tn-Seq. MHBII was used as it is the standard medium for antibiotic resistance testing in clinical laboratories. RPMI+LB was chosen as a second medium because it has been proposed as an alternative to MHBII and in some cases provides more accurate results during antibiotic testing [81]. The 2 mL cultures were harvested by centrifugation when the OD_600_ reached 1.5. The cell pellets were stored at -80°C until processing for Tn-Seq. The genomic DNA was extracted and prepared as described previously [17]. Samples were submitted to the Tufts University Core Facility for sequencing on a HiSeq 2500 instrument.

### Transposon sequencing data analysis

Transposon sequencing data was split by transposon and sample, trimmed, filtered, and mapped using the Galaxy software suite as previously described [17, 21, 82, 83]. A workflow for the processing is provided on GitHub. The resulting SAM files were converted into tab-delimited hop count files using Tufts Galaxy Tn-Seq software (http://galaxy.med.tufts.edu/) or custom python scripts, likewise provided on GitHub, and then converted further into IGV-formatted files, as previously described [17, 21].

Chromosome nucleotide FASTA files for NCTC 8325 (NC_007795.1—HG003 parent strain, as the HG003 genome is not closed), USA300-TCH1516 (NC_010079.1), MSSA476 (NC_002953.3), MW2 (NC_003929.1), and MRSA252 (NC_002952.2) were downloaded from the NCBI genomes database. The genomes were reannotated via Prokka and the pangenome was aligned with Roary, splitting by homolog and using a 90% ID cutoff [84, 85]. Roary group names were then adjusted based on common *S*. *aureus* pangenome gene names found on AureoWiki [86].

Genes in each strain were labeled as essential, non-essential, or uncertain using the TRANSIT software Gumbel method [87]. Only the transposon constructs with transcriptional terminators were included (four of six transposon constructs). For each TRANSIT Gumbel run, data files for each transposon construct in both replicates (for a total of eight files) were submitted and the mean replicates parameter was chosen. Permutation tests with 20,000 permutations were then conducted between each pair of strains for each gene to determine whether differences in essentiality were significant, using the sum of the same data sets used in the Gumbel analysis. Data were normalized by average reads per TA site with reads (non-zero means normalization) and the p-values from each file pair were corrected for multiple hypothesis testing using the Benjamini-Hochberg method. A gene was considered significantly different between two strains if the q-value was less than 0.05. The list of genes essential for all five strains was then analyzed using the online gene ontology tool PANTHER version 13.1, comparing the NCTC 8325 locus tags for the essential genes to the *S*. *aureus* reference gene list and using default settings for the overrepresentation test [88].

Genes depleted or enriched under daptomycin stress were identified by normalizing the treated sample data by non-zero means and performing a Mann-Whitney U test for each gene. The p-values were then corrected for multiple hypothesis testing by the Benjamini-Hochberg method. A depleted gene was considered to be a hit if there were at least 100 reads in the control file, the q-value was less than 0.05, and the treated:control read ratio was less than 0.1. An enriched gene was considered to be a hit if there were at least 100 normalized reads in the treated file, the q-value was less than 0.05, and the read ratio was greater than ten. We then selected one representative daptomycin concentration for each strain in each medium, chosen to have a similar selective pressure (Table 2). Specifically, the highest concentration file with twenty-five or fewer 10-fold depleted genes with a significant q-value was chosen.

**Table 2:** Daptomycin samples included in the multi-strain comparison of depleted and upregulated genes.

Strain	Medium	Concentration (μg/mL)	Depleted Genes
USA300-TCH1516	RPMI+LB	0.12	8
USA300-TCH1516	MHBII	0.5	12
MW2	RPMI+LB	0.25	18
MW2	MHBII	0.5	8
MSSA476	RPMI+LB	0.25	7
MSSA476	MHBII	0.5	8
HG003	RPMI+LB	0.25	9
HG003	MHBII	1	25
MRSA252	RPMI+LB	0.5	19
MRSA252	MHBII	1	18

Genes upregulated under daptomycin stress were identified by a bootstrapping approach. We defined the upstream region (UR) of a gene to be the 500 bp ahead of a gene, based on the 99^th^ percentile of 5’ untranslated regions of transcripts in *E*. *coli* [89]. Only TA sites located in a UR that had at least one read in either the control or the treated data were included in the analysis. Read differences for each site were then calculated by subtracting the control data reads from the treated data reads. For each strand and for each gene, a distribution for the expected difference in UR reads between the treated sample and the control was created by counting the number of qualifying TA sites in the UR (N) and generating 200,000 summed random samples of N read differences from across the genome. A one-sided p-value for each strand was calculated as the proportion of the null distribution values that was greater than the actual value for that gene, and a q-value was obtained using the Benjamini-Hochberg method. We defined an upregulation signature as any UR in which the q-value for the DNA strand matching the gene direction was less than 0.05, there was more than one TA site in the UR, and the opposing strand had a read difference less than the 90^th^ percentile for all URs. Again, only those samples shown in Table 2 were included.

### Whole genome sequence comparisons

To obtain the genomic sequences of the transposon library parent strains, the strains were cultured in MHBII at 37°C shaking overnight. The DNA was harvested using a Promega Wizard Genomic DNA purification kit and cleaned using a Zymo DNA Clean Up Kit. DNA was tagmented using the Illumina Nextera DNA Library Prep kit, but using 1/20^th^ of the volume recommended by the manufacturer and starting DNA concentrations of 0.5, 0.75, 1, and 2 ng/μL. The tagged DNA fragments were then amplified via PCR. The PCR samples contained 11.2 μL of KAPA polymerase mix (Illumina), 4.4 μL each of the 5 μM column and row indexing primers, and 2.5 μL of tagmented DNA. The thermocycler settings were as follows: preincubation (3 min, 72°C), polymerase activation (5 min, 98°C), 13 amplification cycles (denaturation at 98°C for 10 sec, annealing at 62°C for 30 sec, and extension at 72°C for 30 sec), and termination (5 min, 72°C).

To determine which starting DNA concentrations yielded the best fragment size, 3.75 μL of each amplified sample was mixed with 4 μL of 6x loading dye and run on a 1.5% agarose gel at 110 V. The sample with an average length closest to 500 bp was chosen for each strain. Those samples were then cleaned as recommended by Illumina, except that we started with 15 uL of amplified tagmented DNA and 12 μL of AMPure XP beads. The concentrations of the resulting DNA samples were estimated via Qubit Fluorometric Quantification (Thermo Fisher Scientific) following the manufacturer’s instructions. The DNA was then diluted to 1 ng/μL and pooled, and a portion of it was submitted to the Harvard Biopolymer’s Facility for TapeStation and qPCR quality control analysis. Upon passing the quality control step, the DNA was prepared and loaded into the sequencing cartridge as directed by the MiSeq Reagent Kit v3 with 150 cycles (Illumina), including a 1% PhiX control spike-in prepared according to manufacturer’s instructions (PhiX Control v3, Illumina), and paired-end sequenced using a MiSeq instrument.

Genomic data was then analyzed to find SNPs, insertions, and deletions. MetaPhlAn2 was used to verify that the DNA was not contaminated. Sequences were then aligned to the appropriate NCBI reference genome using the Burrow-Wheels Aligner (BWA-MEM, v7.12) with default settings [90]. Note that NCTC 8325 was used for HG003, as the HG003 genome on NCBI is not closed. Duplicate reads were marked with Picard, and Pilon (v1.16) was used for variant calling, with a minimum depth of 1/10^th^ of the average read depth and a minimum mapping quality of 30, unless the average read depth was less than 100, in which case the minimum mapping quality was set to 15 [91, 92]. The resulting VCF files were then evaluated for mutations in relevant areas of the genome (*i*.*e*. the LTA pathway or known *ltaS* suppressors). We compared the predicted protein sequences for LTA pathway members and *ltaS* suppressors in the five strains using the online Clustal Omega tool from EMBL-EBI [93].

### Gene deletion and complementation

To make an anhydrotetracycline (Atet) inducible construct of *ugtP*, a fragment containing the *ugtP* and *ltaA* operon (*SAOUHSC_00953–00952*) and its ribosomal binding site was amplified from HG003 genomic DNA using *ugtP*-F and *ugtP*-R. The fragment was then cloned into pTP63 using KpnI and EcoRI to generate pTP63-*ugtP*, and the pTP63-*ugtP* was transformed into a wild-type RN4220 strain containing pTP44 [94]. Next, the integrated inducible *ugtP* operon was transduced into wild-type HG003, MW2, and USA300-TCH1516 using φ11 phage to generate HG003 P_tet_-*ugtP*, MW2 P_tet_-*ugtP*, and USA300-TCH1516 P_tet_-*ugtP*. To generate inducible *ugtP* strains, the P_tet_-*ugtP* strains were grown in TSB containing 0.3 μM Atet for 6 hours at 30°C, after which they were transduced with a transposon-inactivated *ugtP* marked with an erythromycin resistance gene [13]. The desired mutants were selected on TSB agar containing 5 μg/mL erythromycin and 0.3 μM Atet and confirmed by PCR using the primers *ugtP*-CA and *ugtP*-CB.

To construct an Atet-inducible *ltaA* construct, the *ltaA* gene (*SAOUHSC_00952*) and the *ugtP-ltaA* operon ribosome binding site were amplified from HG003 genomic DNA using primers *ltaA*-F and *ugtP*-R and cloned into pTP63 to generate pTP63-*ltaA*. As described above, pTP63-*ltaA* was transduced into wild-type HG003, MW2, and USA300-TCH1516, and the resulting strains were then transduced with a Δ*ltaA* construct marked with a kanamycin resistance gene to generate the inducible *ltaA* strains [95].

To construct an Atet-inducible *ltaS* construct, the *ltaS* gene and its ribosome binding site were PCR amplified using the primers *ltaS*-F and *ltaS*-R and cloned into pTP63 to make pTP63-*ltaS*. To make an inducible *ltaS* strain, pTP63-*ltaS* and then a Δ*ltaS* construct marked with an erythromycin resistance gene were transduced into wild-type HG003, MW2, and USA300-TCH1516 as described above [44]. The *ltaS* mutants were confirmed by PCR using the primers *ltaS*-CA and *ltaS*-CB.

To construct the *clpX* construct, the *clpX* gene and its native promoter were PCR amplified from RN4220 genomic DNA using the primers *clpX*-f-BamHI and *clpX*-r-H6. A kanamycin resistance gene was PCR amplified using the primers Kan-f-H6 and Kan-r-SalI. The two fragments were joined together using overlap PCR using the primers *clpX*-f-BamHI and Kan-r-SalI. The resulting *clpX*-Kan fragment was cloned into the low-copy *S*. *aureus* expression plasmid pLOW using SalI and BamHI to generate pLOW-*clpX*. Next, pLOW-*clpX* was transformed into wild-type RN4220 and transduced using φ85 phage into wild-type MW2 and MW2 with an Atet-inducible *ltaS* construct to generate MW2 P-*clpX* and MW2 P_tet_-*ltaS* P-*clpX*. The *clpX* construct was confirmed by PCR using the primers pLOW-f and pLOW-r.

Strain construction has previously been described for several mutants including RN4220 Δ*vraFG* [96], HG003 Δ*ezrA* [95], and HG003 *SAOHSC_02149*::tn [21]. The *gpsB* transposon mutant from the Nebraska transposon library [13] was transduced into wild-type HG003 using φ85 phage to generate HG003 *gpsB*::tn. Previously described allelic replacement approaches were used to create HG003 Δ*SAOUHSC_01050* [95] as well as the *graR* and *mprF* deletions [96] which were transduced into HG003 using φ85 phage.

### Spot dilution assays

Overnight cultures were diluted in TSB and grown at 30°C until mid-log phase, then diluted to an OD_600_ of 0.1. Five 10-fold dilutions of the resulting cultures were prepared, and 5 μL of each dilution was spotted on TSB plates with or without 0.3 μM Atet inducer and, where indicated, daptomycin. We found that calcium addition was not needed for daptomycin activity in TSB agar. Plates were imaged after up to 16 hours of incubation. For the *ltaS* experiments, plates were incubated at 30°C, 37°C, or 42°C as indicated. For the spot dilution assays in the presence and absence of daptomycin, plates were incubated at 37°C.

### Growth curves

Overnight cultures were diluted in MHBII and grown at 37°C until mid-log phase, then diluted to an OD_600_ of 0.001 in MHBII supplemented with 0.5 mM CaCl_2_. The resulting cultures were added to 96-well plates containing a 2-fold titration of daptomycin concentrations ranging from 16 to 0.125 μg/ml. Plates were incubated with shaking at 37°C for 20 hours. The OD_600_ in each well was measured in 20-minute intervals and background was subtracted using media-only wells.

### LTA western blot

LTAs from MW2, MW2 P_tet_-*ltaS*, and 4S5, a strain derived from RN4220 known to not produce LTAs [44], were isolated and detected via western blot using a procedure similar to that previously described [97]. Overnight cultures of each strain were grown shaking at 30°C in TSB supplemented with 7.5% NaCl, with MW2 P_tet_-*ltaS* grown both in the presence and absence of 0.4 μM Atet. Cultures were then diluted 1:50 in the same medium and incubated shaking at 30°C until the OD_600_ was between 0.6 and 0.75. The equivalent of 1 mL of OD_600_ 0.8 was then harvested from each sample by centrifuging at 8000 x *g* for three minutes. The cell pellets were resuspended in 50 μL of a buffer composed of 50 mM Tris pH 7.5, 150 mM NaCl, and 200 μg/mL lysostaphin. Samples were incubated at 37°C for 10 minutes. Then, 50 μL of 4x SDS-PAGE loading buffer was added and the samples were boiled for 30 minutes. After returning the samples to room temperature, 100 μL water and 0.5 μL of proteinase K (New England Biolabs) was added to each sample, and samples were then incubated at 50°C for 2 hours. After cooling samples to room temperature, 2 μL of AEBSF was added to each sample to quench the proteinase K. 10 μL of each sample and 5 μL of Precision Plus Protein Dual Xtra standard (BioRad) were loaded onto a 4–20% TGX precast gel (BioRad) and the gel was run for 30 minutes at 200 V using a running buffer composed of 0.5% Tris, 1.5% glycine, and 0.1% SDS. Proteins were transferred to a methanol-activated PVDF membrane using a TransBlot Turbo (BioRad) via the pre-installed mixed molecular weight setting. The membrane was rinsed with TBST (0.05% Tween-20) and incubated rocking overnight at 4°C in 5% milk in TBST to block. After washing the membrane with TBST three times for five minutes each, the LTAs were bound with a 1:750 solution of a mouse anti-LTA antibody (Hycult Biotech) in TBST for 45 min. The membrane was then washed an additional three times for five minutes each before incubating in a 1:2000 dilution of anti-mouse horseradish peroxidase conjugated antibody (Cell Signaling Technologies) in TBST. The membrane was washed a final five times for five minutes each and then exposed to ECL western blotting substrate (Pierce). The membrane was imaged on a FluoroChem R gel doc (ProteinSimple) with a 2 minute 40 second exposure for luminescence to detect the LTAs and automatic exposure for red and green fluorescence channels to detect the protein standard.

## Supporting information

S1 Fig Genes shared among strains. A matrix showing the number of genes shared between pairs of strains according to analysis with the Roary software package. The number shared between a strain and itself (*e*.*g*., HG003 and HG003) represents the full genome of that strain. Each cell of the matrix is colored on a continuum based on the number of genes, with the lowest value (2259 genes shared between MRSA252 and MSSA476) set to white and the highest value (2706 genes in the MRSA252 genome) set to the darkest shade of green.
(PDF)

S2 Fig Gene essentiality varies by strain. The number of essential genes per strain (horizontal bars) and the number of genes found to be essential for all five or a subset of the *S*. *aureus* strains (vertical bars, with black dots denoting strains for which the gene is essential).
(PDF)

S3 Fig Tn-Seq data reveals variable dependence on the lipoteichoic acid pathway among *S*. *aureus* strains. Each column of graphs represents a gene in the LTA pathway, with rows representing strains. Reads in the Tn-Seq data, expressed on a log_10_ scale, are plotted against the position in the genome indicated on the x-axis with a depiction of the gene context underneath each plot. The y-axis is truncated to 500 reads. Purple lines indicate plus-strand reads. Teal lines indicate minus-strand reads.
(PDF)

S4 Fig MW2Δ*ltaS* mutants are temperature sensitive. Growth on agar plates of wild-type (WT) and *ltaS* complementation strains for HG003, MW2, and USA300-TCH1516 with and without inducer at 30°C, 37°C, and 42°C.
(PDF)

S5 Fig Expression of the HG003 *clpX* allele causes a decrease in fitness of MW2Δ*ltaS*. Growth on agar plates of MW2 wild-type (WT) and *ltaS* complementation strains in the presence and absence of the HG003 *clpX* allele with and without inducer at 30°C and 37°C.
(PDF)

S6 Fig Genes identified in Tn-Seq are important for survival in the presence of daptomycin. Growth on agar plates of wild type (WT) and mutants in the presence or absence of daptomycin at 37°C. For strains in the RN4220 background, 2 μg/mL daptomycin was used; in the HG003 background, 2.5 μg/mL daptomycin was used. Hypothetical genes are annotated according to their NCTC 8325 locus tag numbers.
(PDF)

S7 Fig *ltaA* is important for survival in USA300-TCH1516 and MW2 in the presence of daptomycin. Growth on agar plates of wild-type, Δ*ltaA*, and inducible *ltaA* complementation strains for MW2 and USA300-TCH1516 in the presence or absence of 2.5 μg/mL daptomycin at 37°C.
(PDF)

S8 Fig *ltaA* mutants have delayed entry into the exponential phase of growth in the presence of daptomycin. Growth curves for wild-type (WT) and Δ*ltaA* strains for HG003 and MW2 in liquid media in the presence or absence of sub-MIC daptomycin at 37°C. For strains in the HG003 background, 2 μg/mL daptomycin was used; in the MW2 background, 1 μg/mL daptomycin was used. Each condition was replicated at least 3 times and a single growth curve representative of the trend is shown.
(PDF)

S1 Table Comparison of essential genes in *S*. *aureus* strains. A table of all genes that are essential for at least one strain, with essentiality designations for each gene in each strain for the TRANSIT Gumbel analysis and the subsequent permutation test which was used to determine whether differences between strains seen in the Gumbel results were significant. If differences were not significant, essential (E) and nonessential (NE) designations were converted to uncertain (U). Genes are sorted descending by the number of strains for which the gene is essential.
(XLSX)

S2 Table Core essential genes in *S*. *aureus*. A list of the genes that were determined to be essential in all five *S*. *aureus* strains. Two tables are provided, one being a more conservative list in which all genes had to be listed as essential in all strains and the other in which each gene had to have at least one essential (E) designation and no non-essential (NE) designations (*i*.*e*. includes those genes that are essential in some strains and indeterminate (U) in others). It should be noted that some genes that are known to be essential (*e*.*g*., *murG*) are listed in the latter list and not the former. The Tn-Seq-based gene essentiality designations here depend on the ability of a strain to receive a transposon from a phage, grow on a plate, and then grow in culture in competition with other mutants. Thus, some genes, such as *tarO*, are labeled as essential due to plating defects rather than true essentiality.
(XLSX)

S3 Table Gene ontology analysis reveals central dogma pathways to be universally essential in *S*. *aureus*. Lists the results from performing gene ontology analysis on the genes that were essential in all five strains, using the more conservative list from the S2 Table. Red text highlights those processes mentioned in the main text. DNA, RNA, and protein metabolic processes are significantly over-represented among the genes essential for all five strains of *S*. *aureus* studied. Phospholipid and monosaccharide synthesis were likewise over-represented, while genes of unknown function were under-represented. Processes with a corrected p-value of 0.05 or greater are grayed out.
(XLSX)

S4 Table Genes depleted under daptomycin exposure. A table of the genes that were 10-fold depleted of reads in the normalized daptomycin file compared to the control file with q-values less than 0.05 and at least 100 reads in the control file.
(XLSX)

S5 Table Genes upregulated under daptomycin exposure. A table of the genes that had upregulation signatures under daptomycin exposure. See Methods for details of analysis.
(XLSX)

S6 Table Genes enriched under daptomycin exposure. A table of the genes that were 10-fold enriched in reads in the normalized daptomycin file compared to the control file with q-values less than 0.05 and at least 100 normalized reads in the daptomycin file.
(XLSX)

S7 Table Primers, plasmids, and strains used in this study. (XLSX)
