# Supplementary material for: Multi-strain Tn-Seq reveals common daptomycin resistance determinants in Staphylococcus aureus
Source: PLoS Pathog. 2019 Nov 18;15(11):e1007862. doi: 10.1371/journal.ppat.1007862 (PMC6934316; doi:10.1371/journal.ppat.1007862)
Supplement: S1 Fig — A matrix showing the number of genes shared between pairs of strains according to analysis with the Roary software package. The number shared between a strain and itself (e.g., HG003 and HG003) represents the full genome of that strain. Each cell of the matrix is colored on a continuum based on the number of genes, with the lowest value (2259 genes shared between MRSA252 and MSSA476) set to white and the highest value (2706 genes in the MRSA252 genome) set to the darkest shade of green. (PDF) [file ppat.1007862.s001.pdf]

Number of Genes Shared Between Strains

	HG003	USA300	MW2	MSSA476	MRSA252
HG003	2631	2466	2376	2378	2266
USA300		2657	2392	2369	2318
MW2			2565	2484	2267
MSSA476				2545	2259
MRSA252					2706
