# Supplementary figures and images for: Multi-strain Tn-Seq reveals common daptomycin resistance determinants in Staphylococcus aureus
Source: PLoS Pathog. 2019 Nov 18;15(11):e1007862. doi: 10.1371/journal.ppat.1007862 (PMC6934316; doi:10.1371/journal.ppat.1007862)

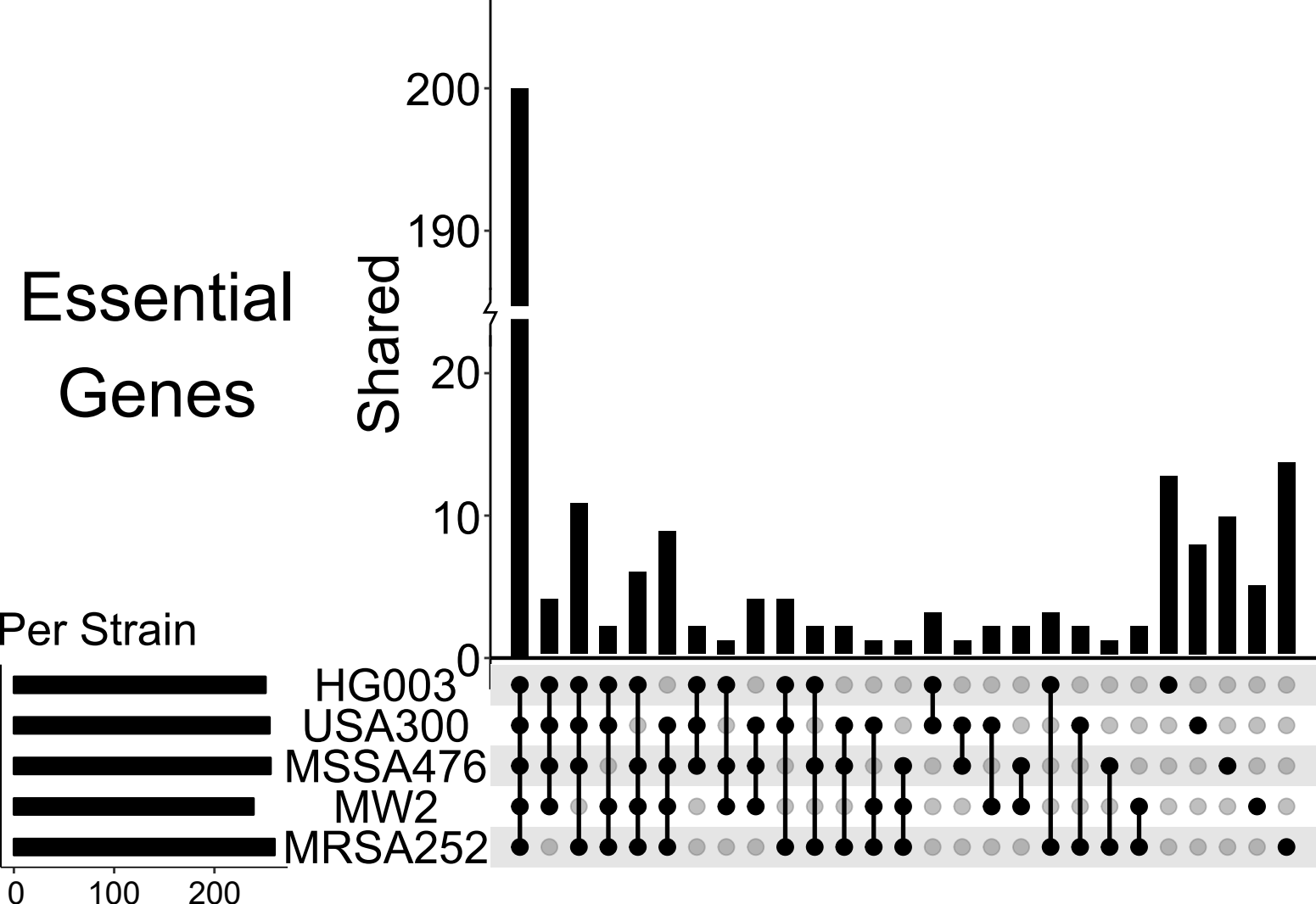

Supplement: S2 Fig — The number of essential genes per strain (horizontal bars) and the number of genes found to be essential for all five or a subset of the S. aureus strains (vertical bars, with black dots denoting strains for which the gene is essential). (PDF) [file ppat.1007862.s002.pdf]

Plus Strand

Minus Strand

*pgcA*

*gtaB*

*ItaA* and *ugtP*

*ItaS*

HG003

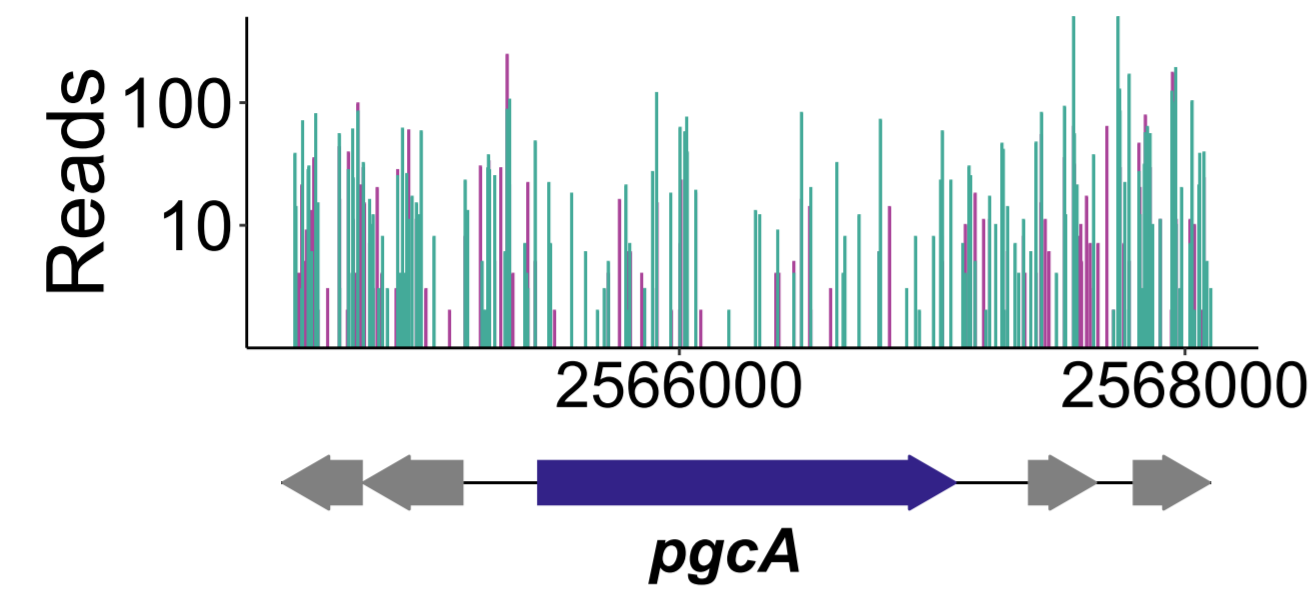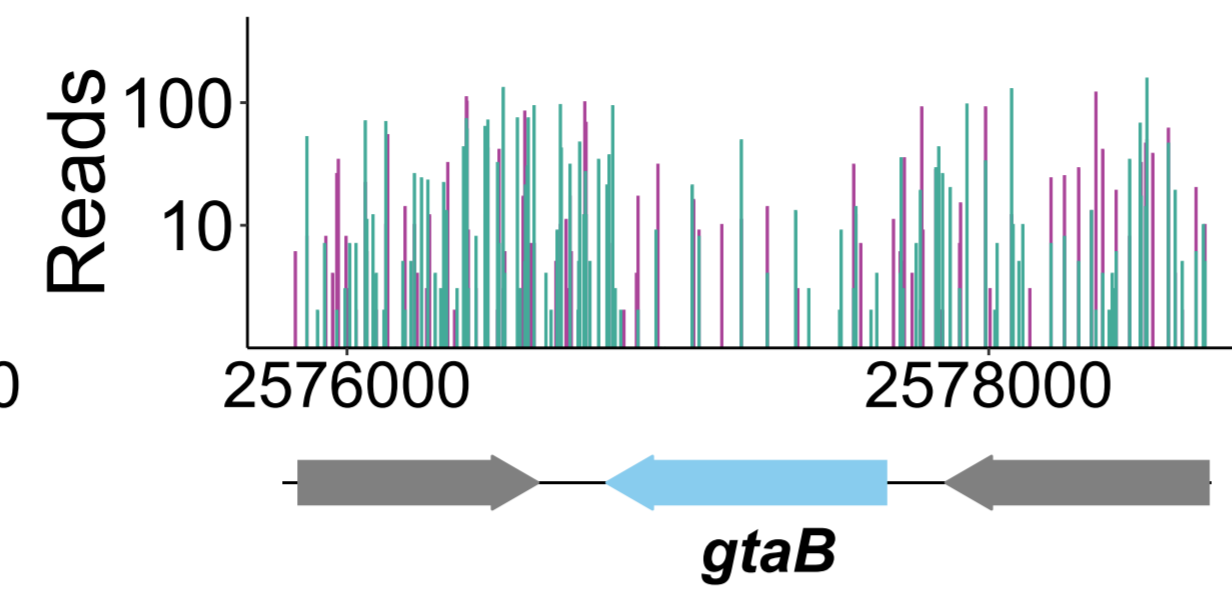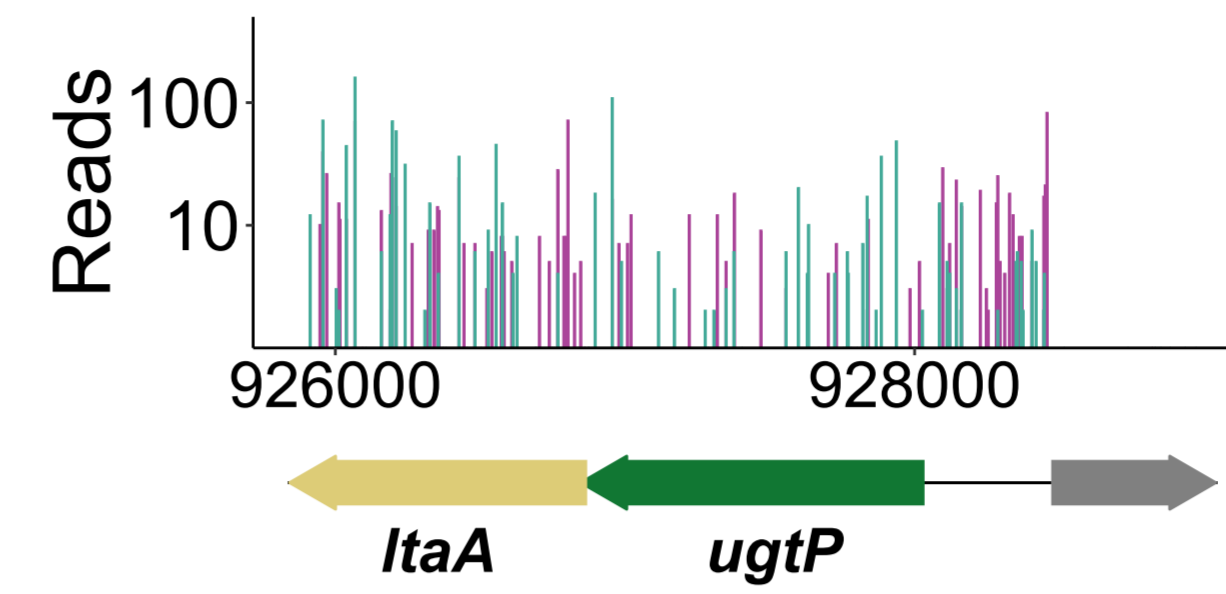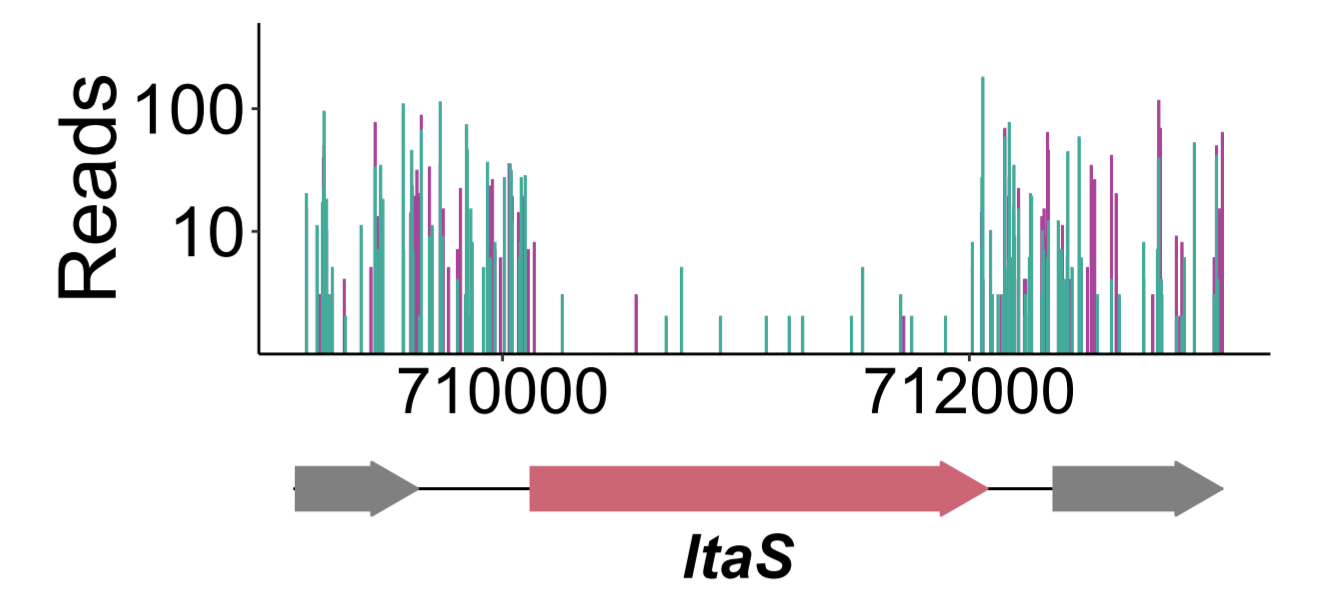

USA300

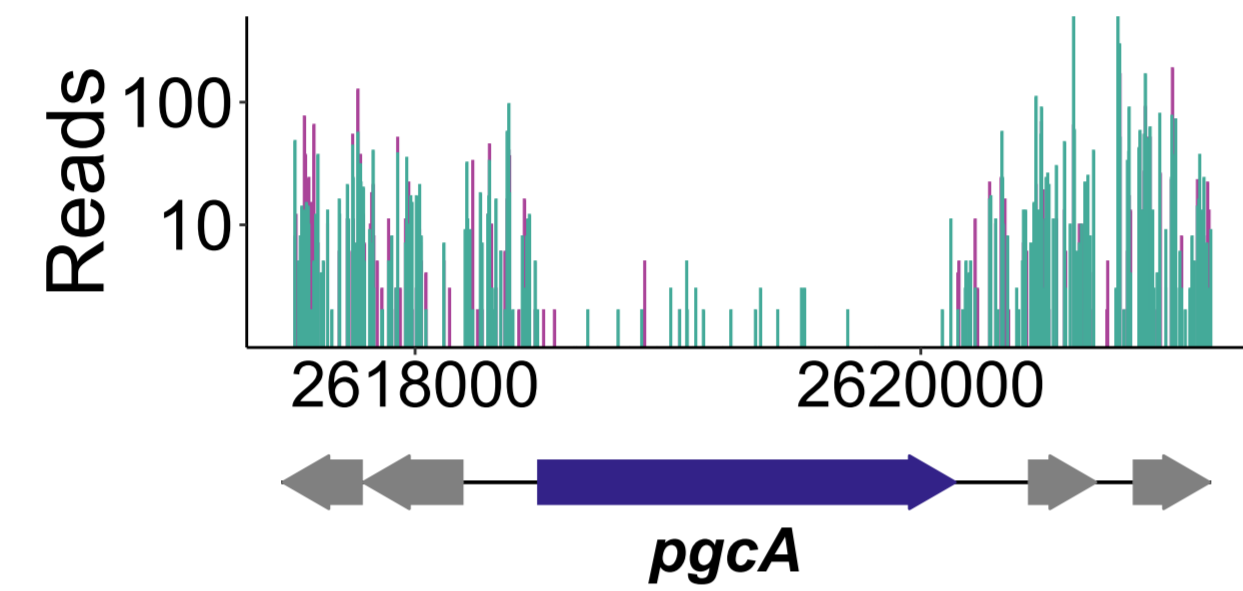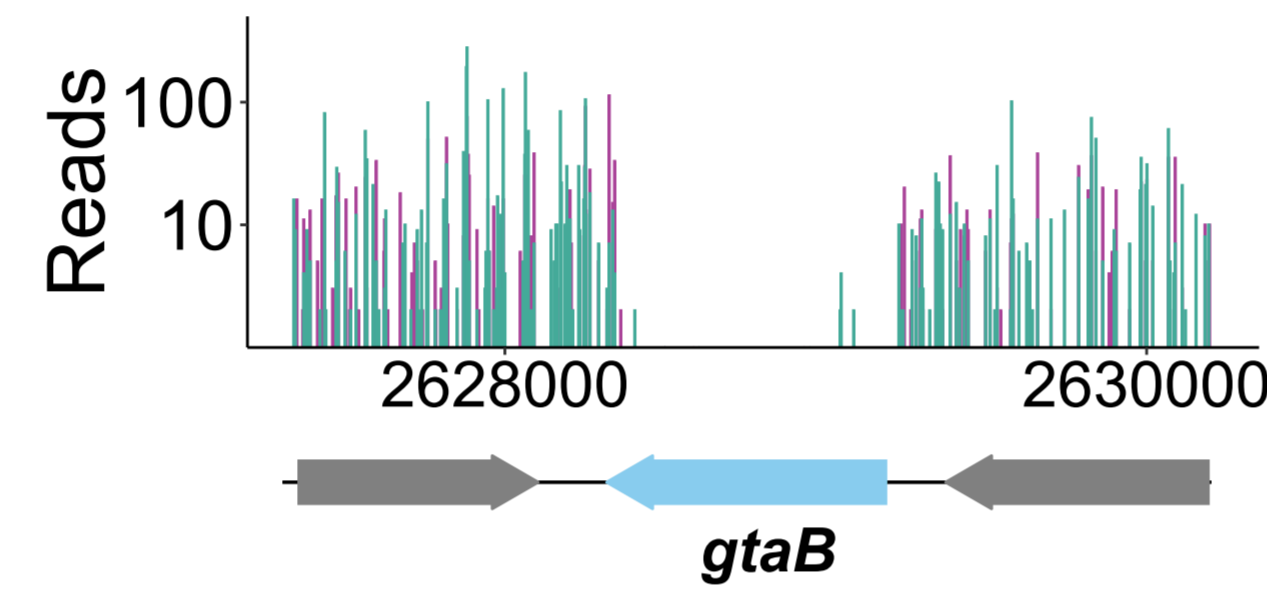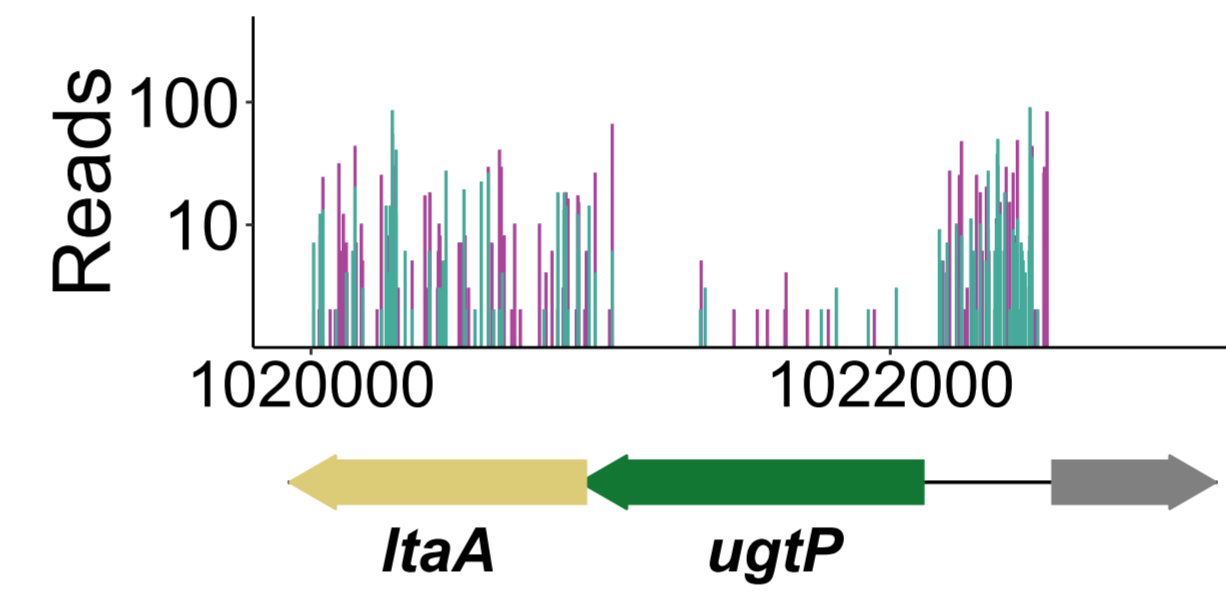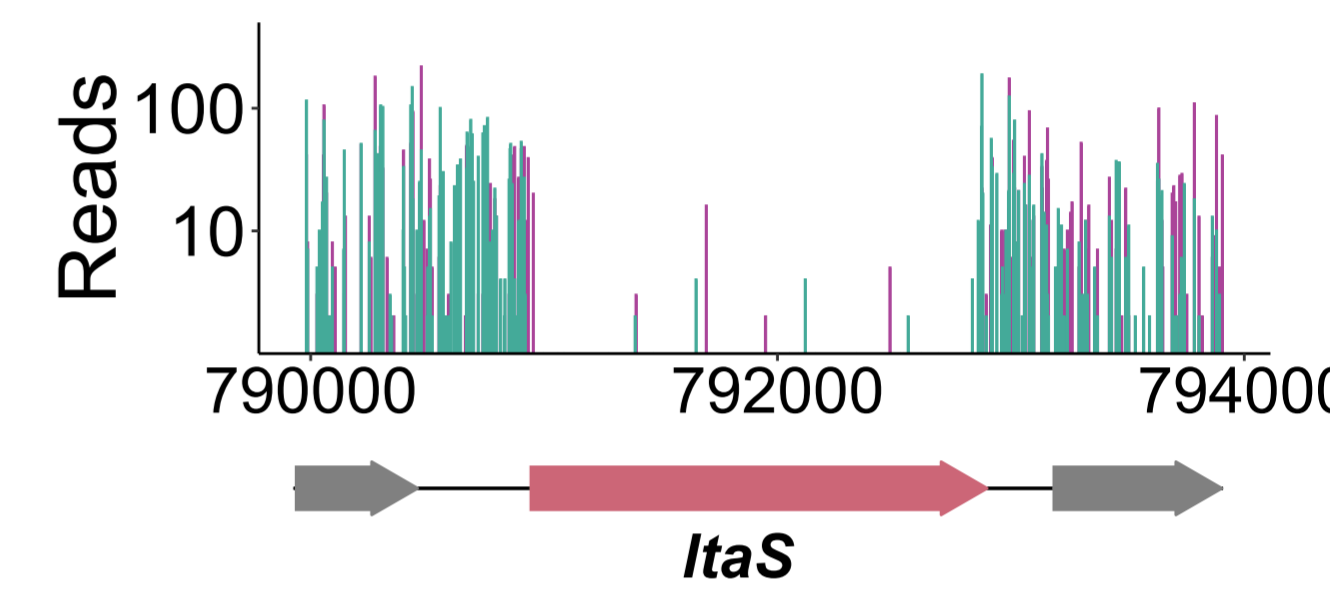

MSSA476

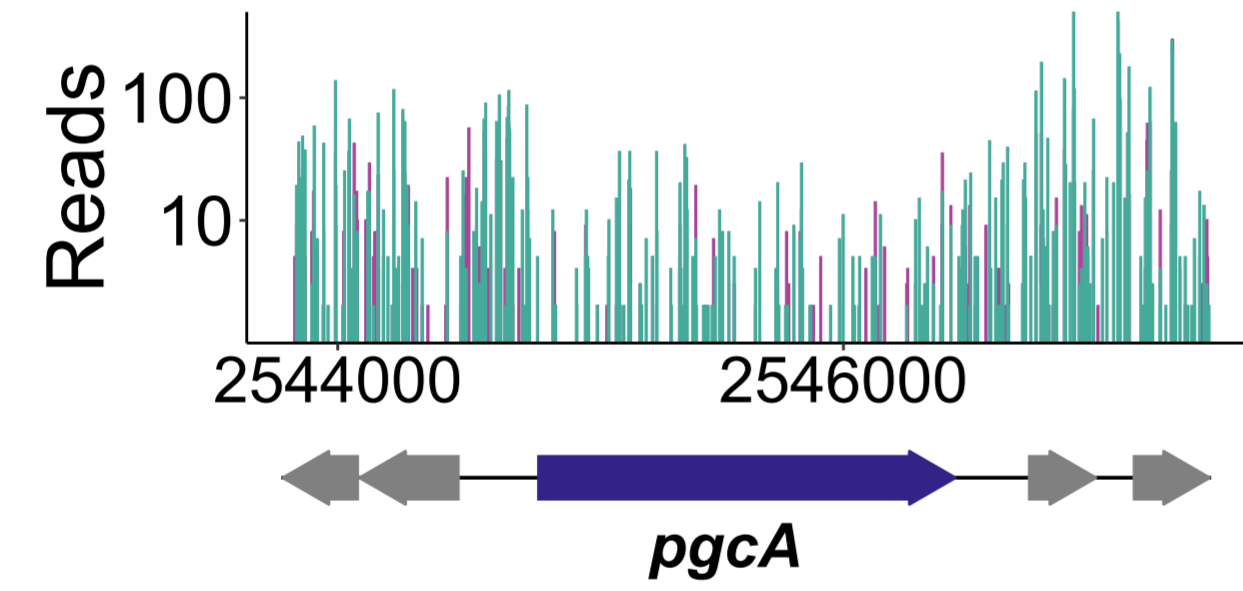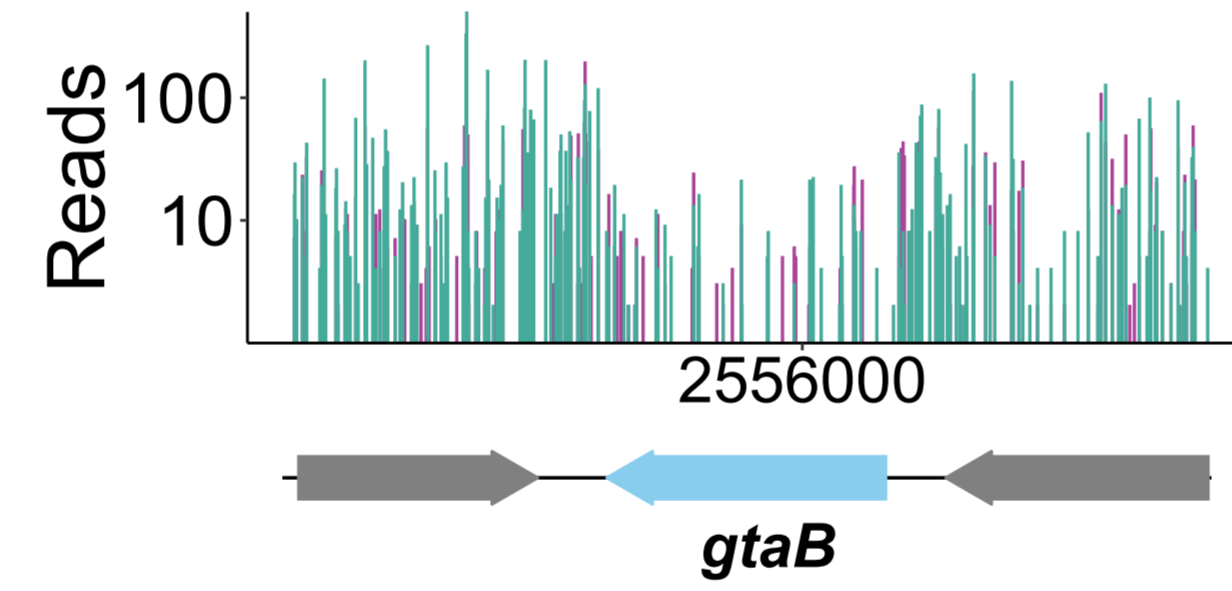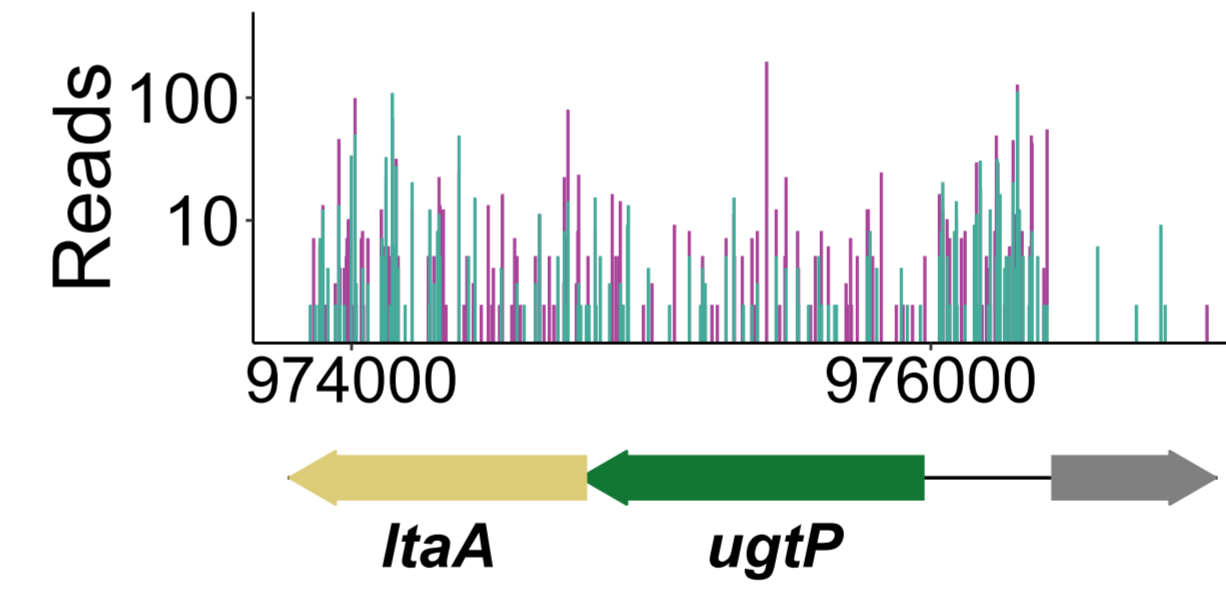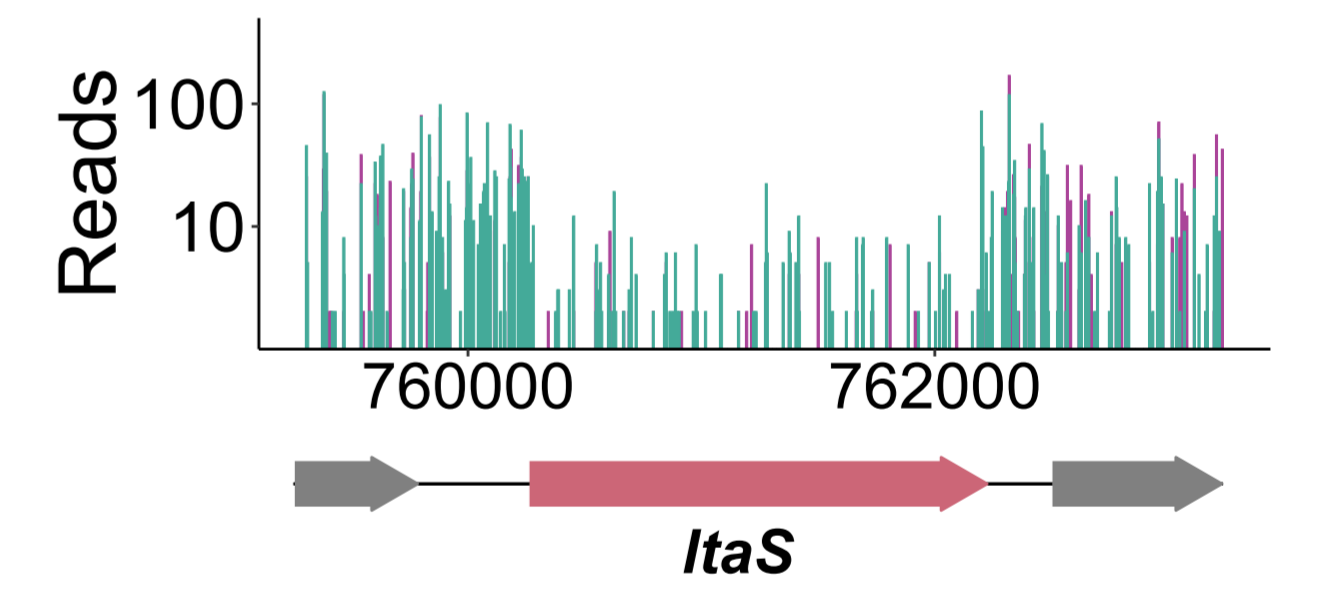

MW2

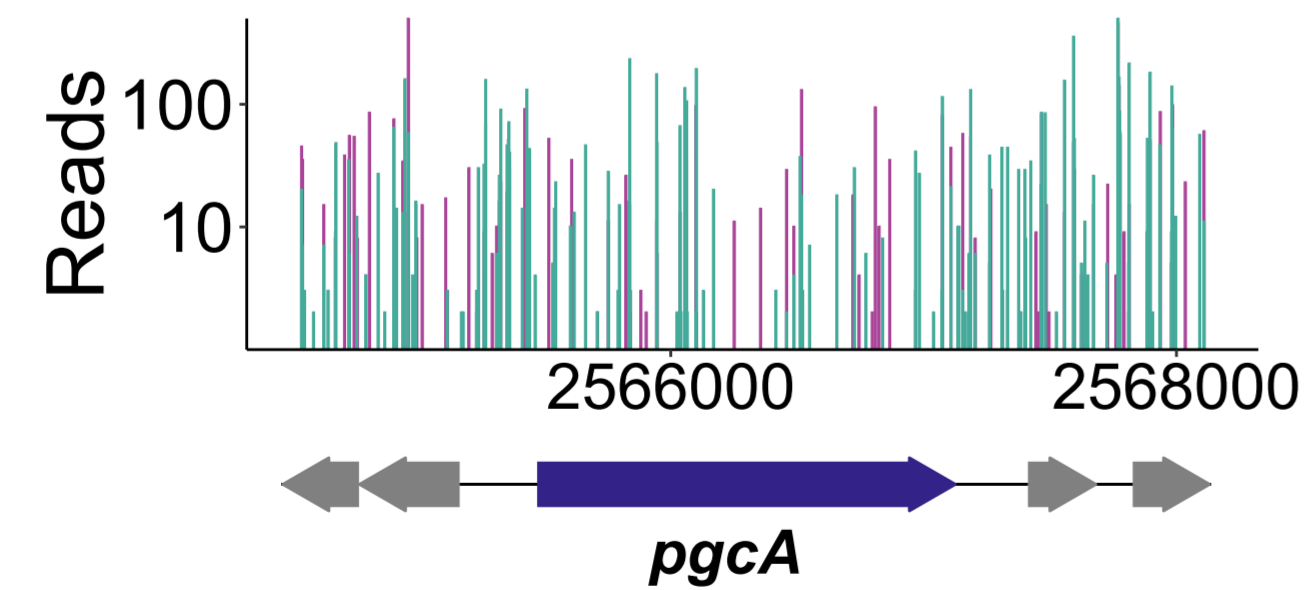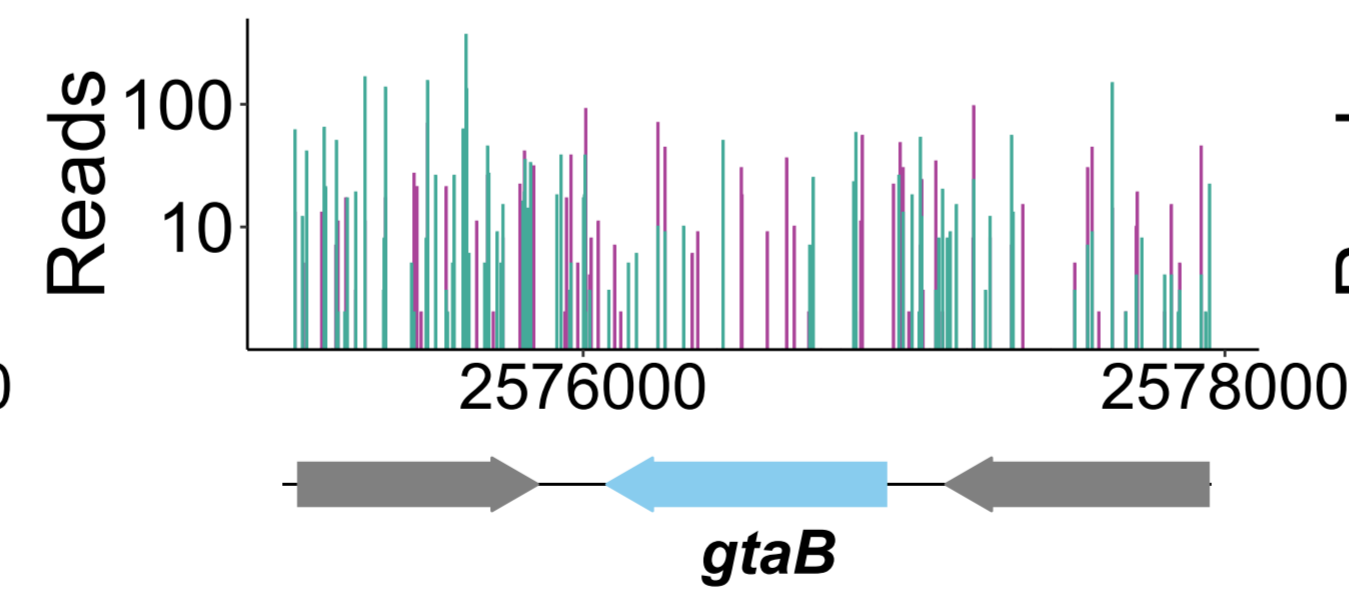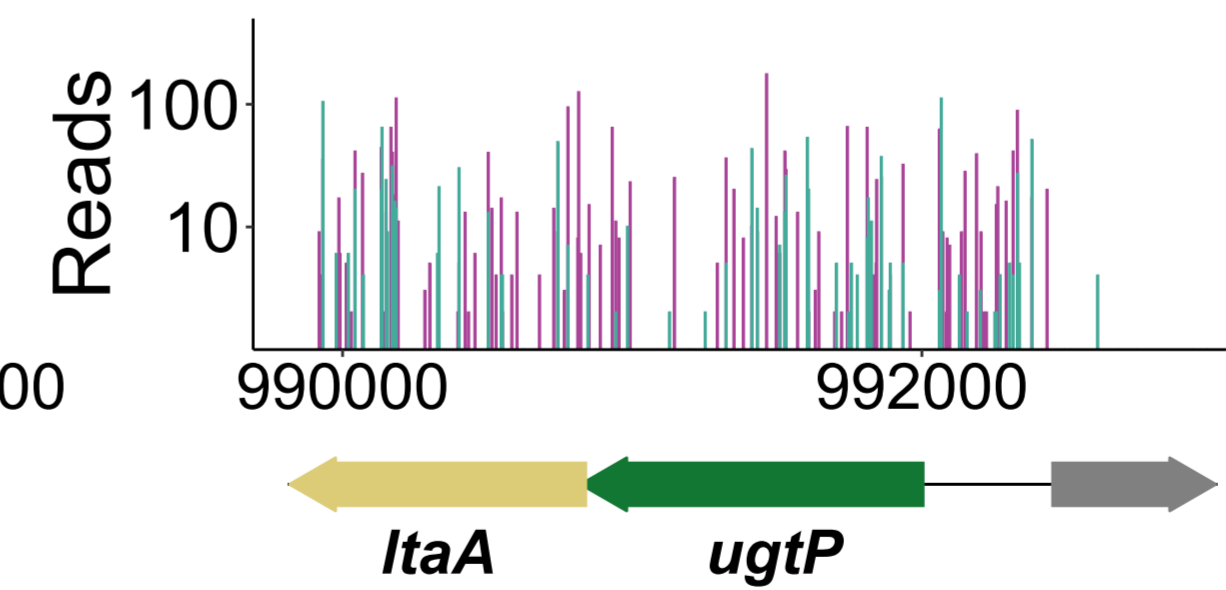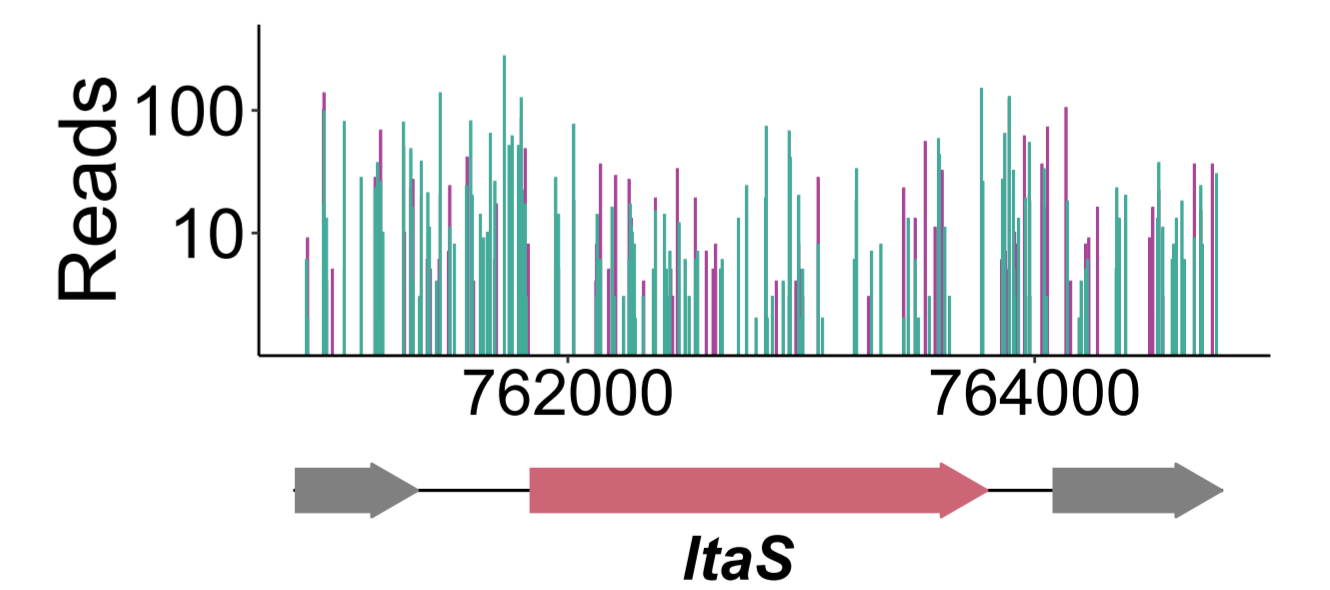

MRSA252

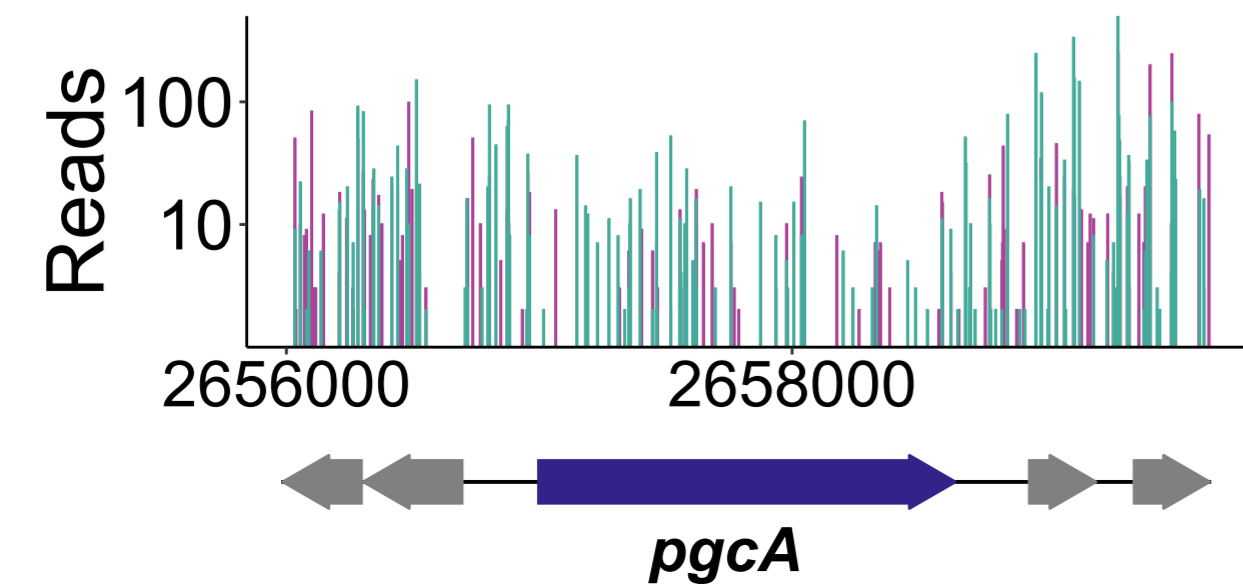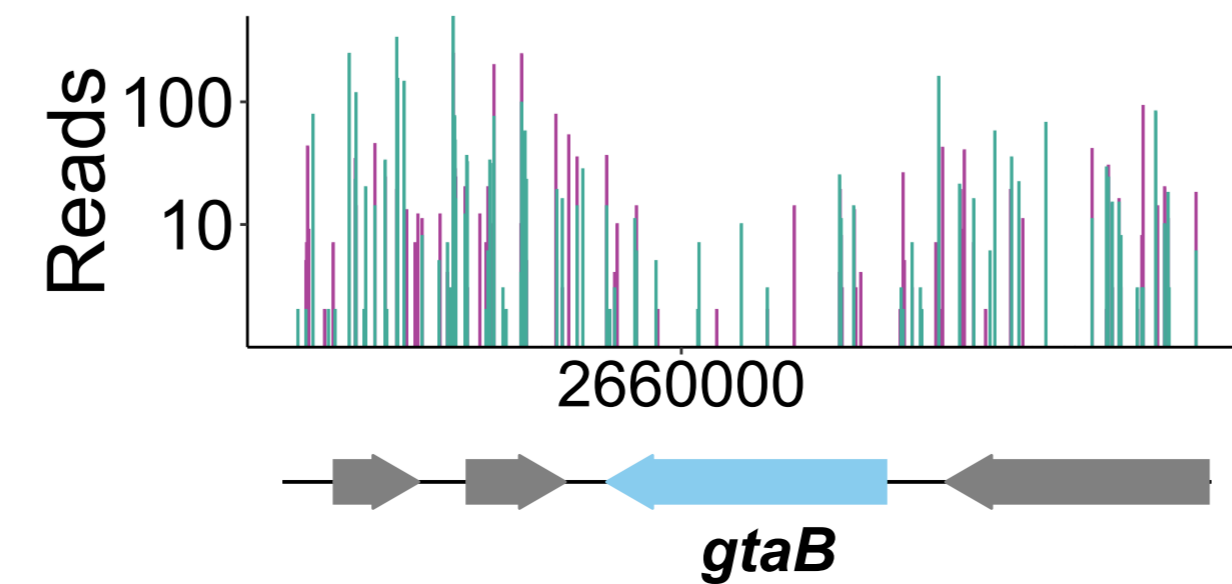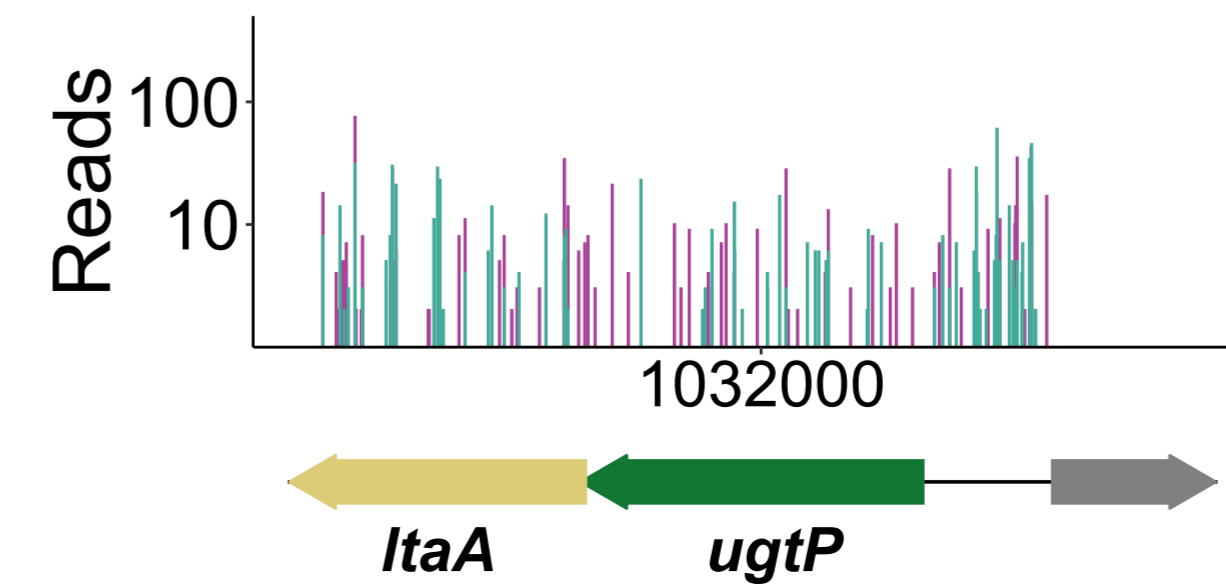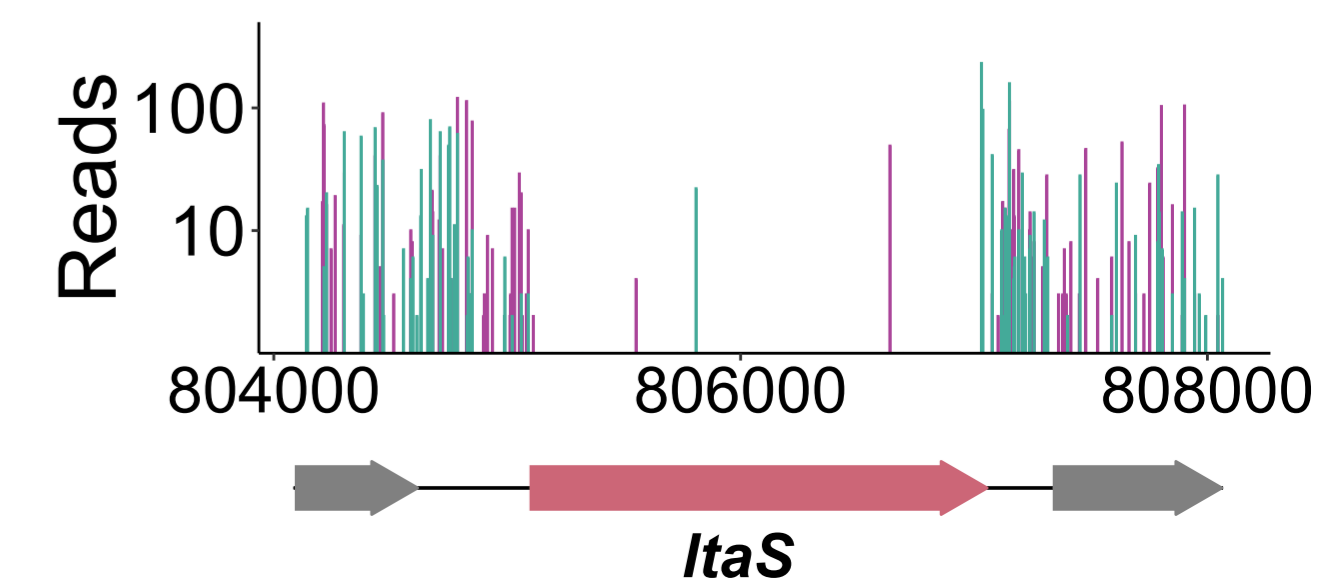

Supplement: S3 Fig — Each column of graphs represents a gene in the LTA pathway, with rows representing strains. Reads in the Tn-Seq data, expressed on a log10 scale, are plotted against the position in the genome indicated on the x-axis with a depiction of the gene context underneath each plot. The y-axis is truncated to 500 reads. Purple lines indicate plus-strand reads. Teal lines indicate minus-strand reads. (PDF) [file ppat.1007862.s003.pdf]

+ Inducer

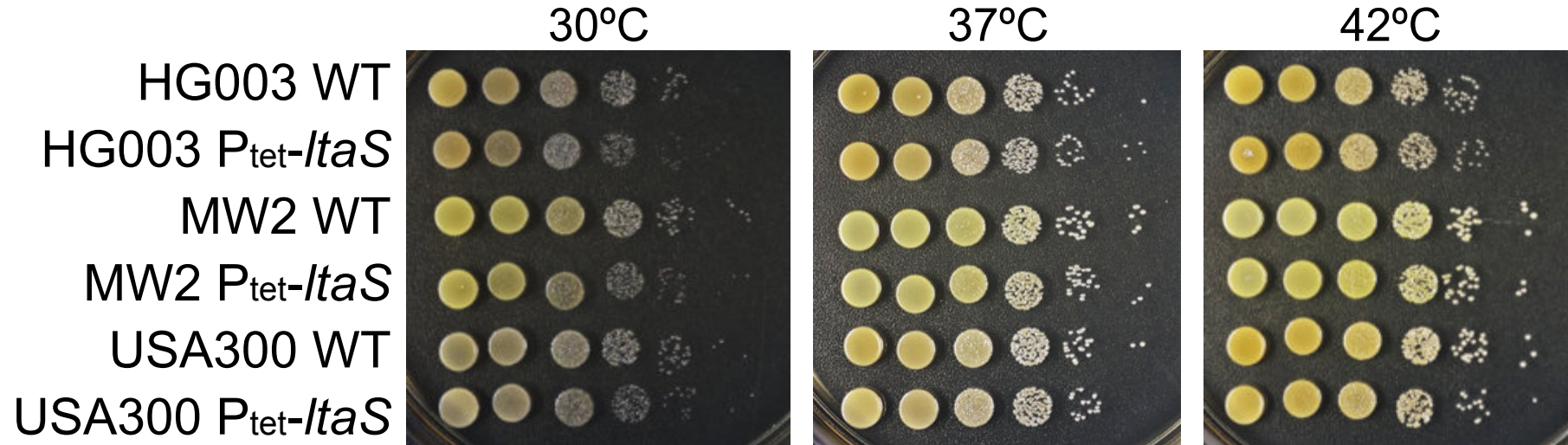

- Inducer

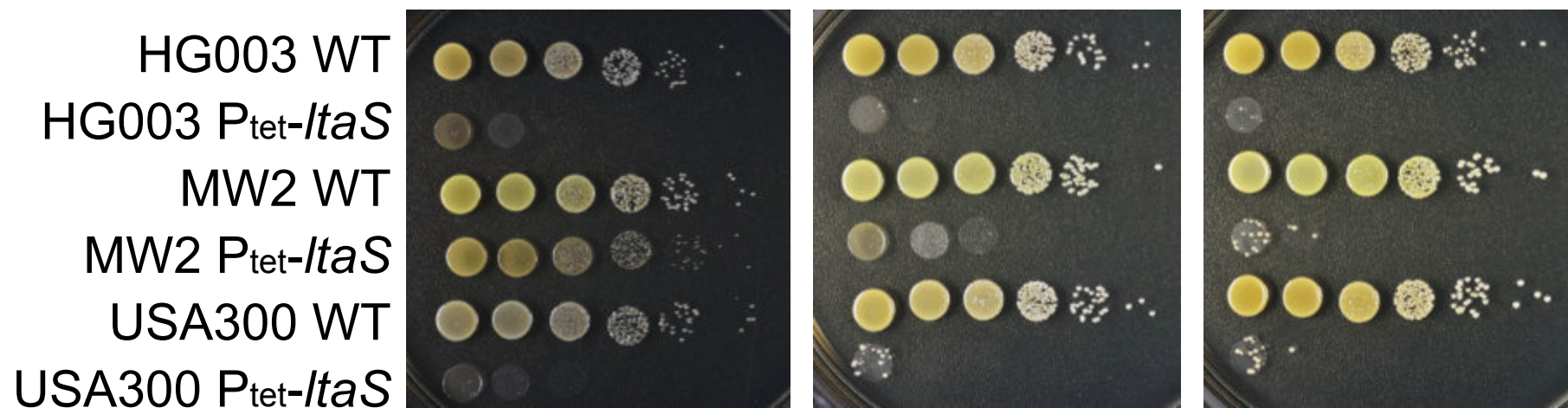

Supplement: S4 Fig — Growth on agar plates of wild-type (WT) and ltaS complementation strains for HG003, MW2, and USA300-TCH1516 with and without inducer at 30°C, 37°C, and 42°C. (PDF) [file ppat.1007862.s004.pdf]

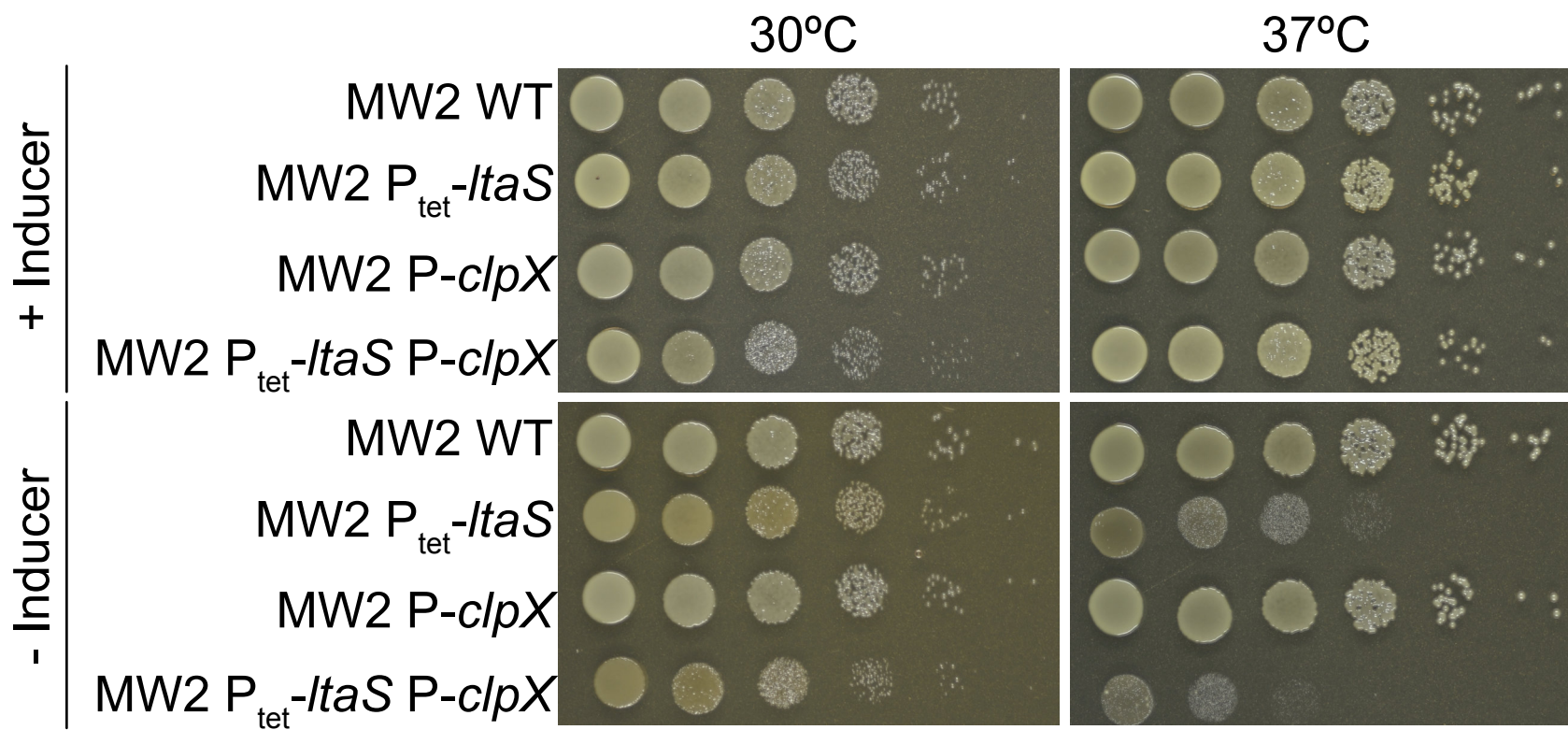

Supplement: S5 Fig — Growth on agar plates of MW2 wild-type (WT) and ltaS complementation strains in the presence and absence of the HG003 clpX allele with and without inducer at 30°C and 37°C. (PDF) [file ppat.1007862.s005.pdf]

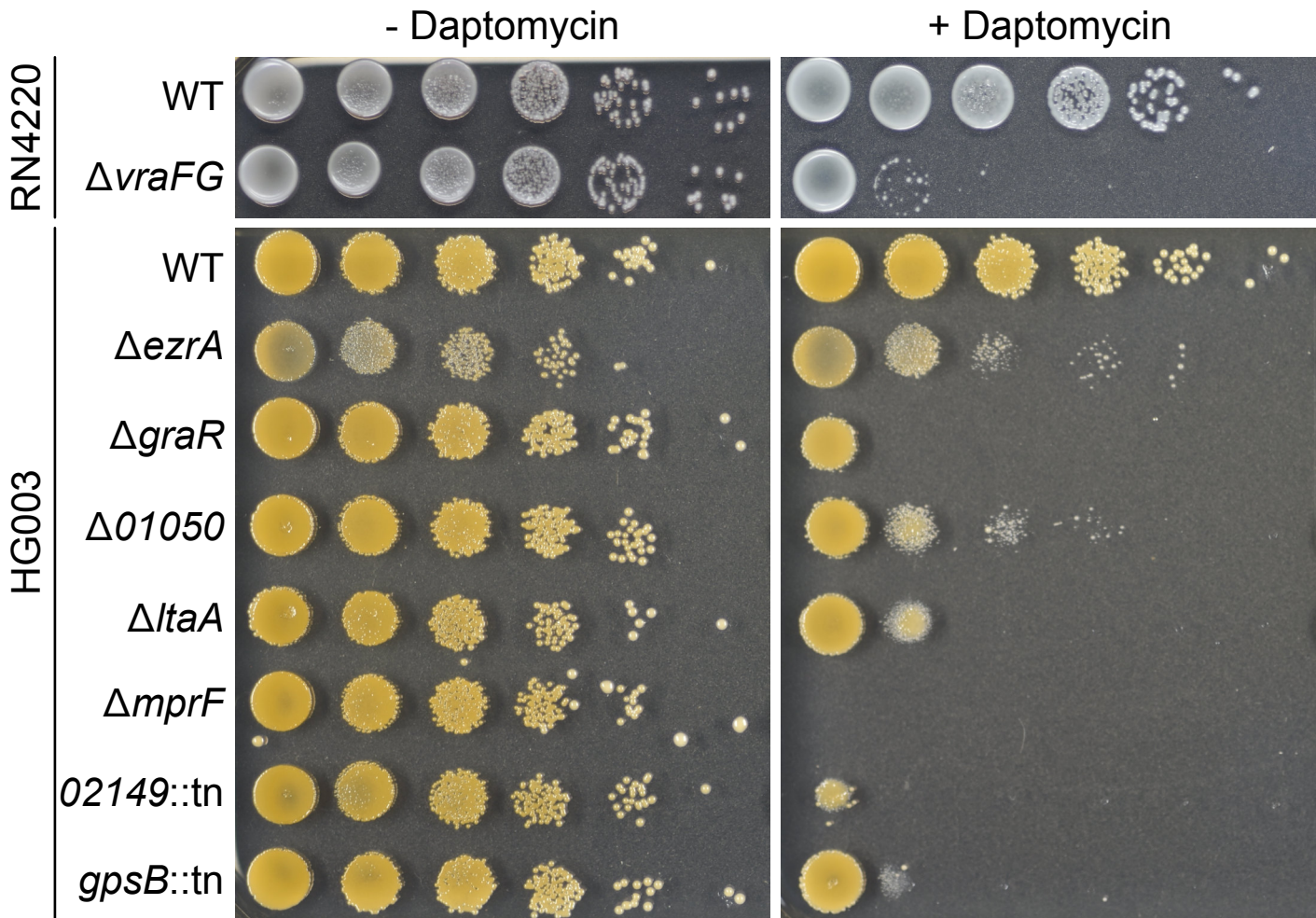

Supplement: S6 Fig — Growth on agar plates of wild type (WT) and mutants in the presence or absence of daptomycin at 37°C. For strains in the RN4220 background, 2 μg/mL daptomycin was used; in the HG003 background, 2.5 μg/mL daptomycin was used. Hypothetical genes are annotated according to their NCTC 8325 locus tag numbers. (PDF) [file ppat.1007862.s006.pdf]

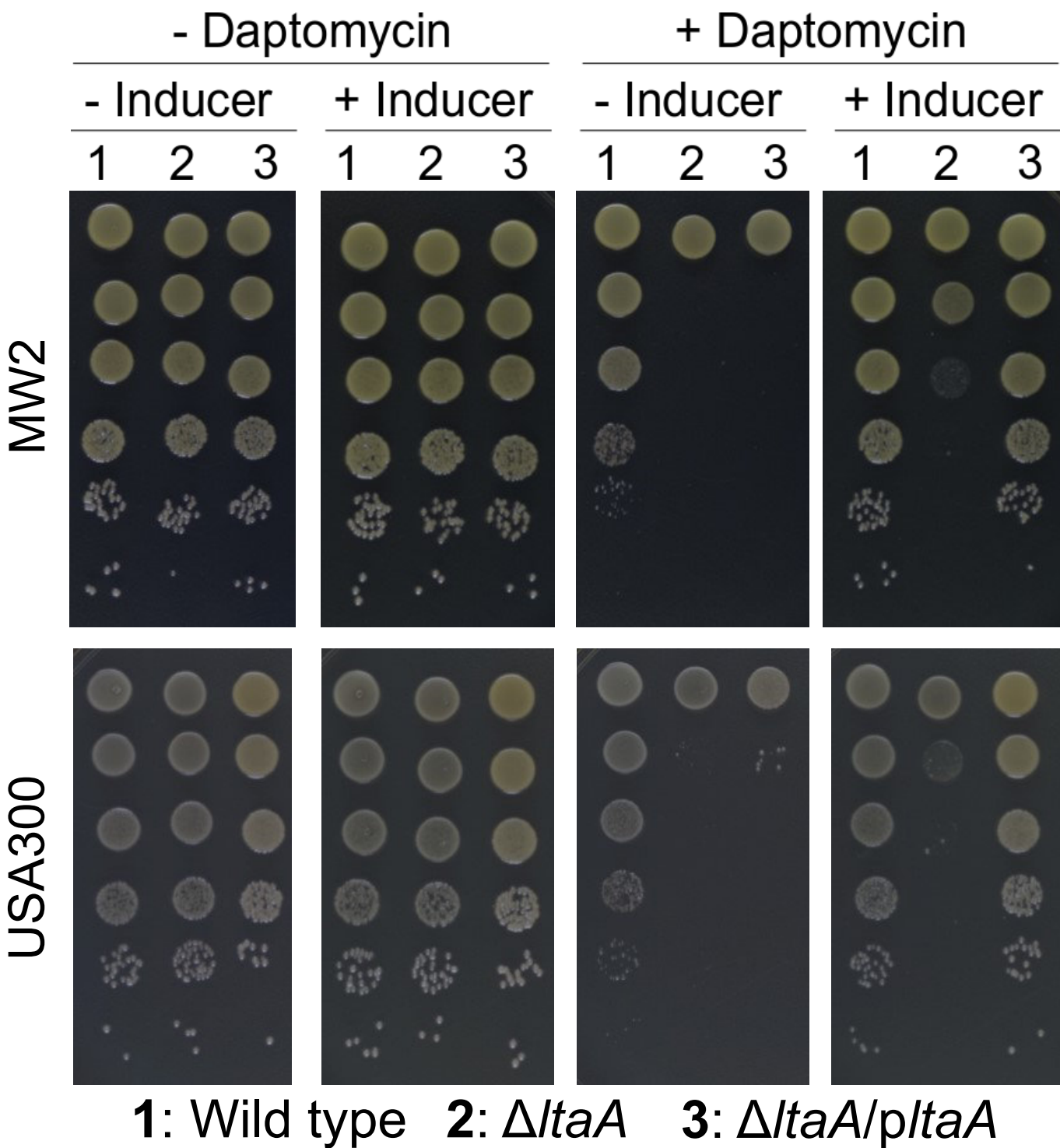

Supplement: S7 Fig — Growth on agar plates of wild-type, ΔltaA, and inducible ltaA complementation strains for MW2 and USA300-TCH1516 in the presence or absence of 2.5 μg/mL daptomycin at 37°C. (PDF) [file ppat.1007862.s007.pdf]

MW2

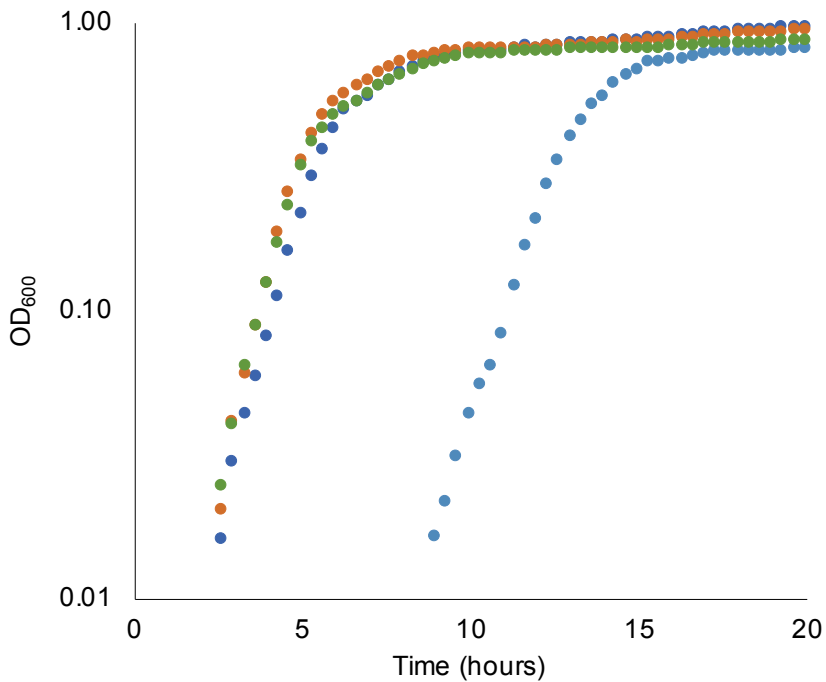

HG003

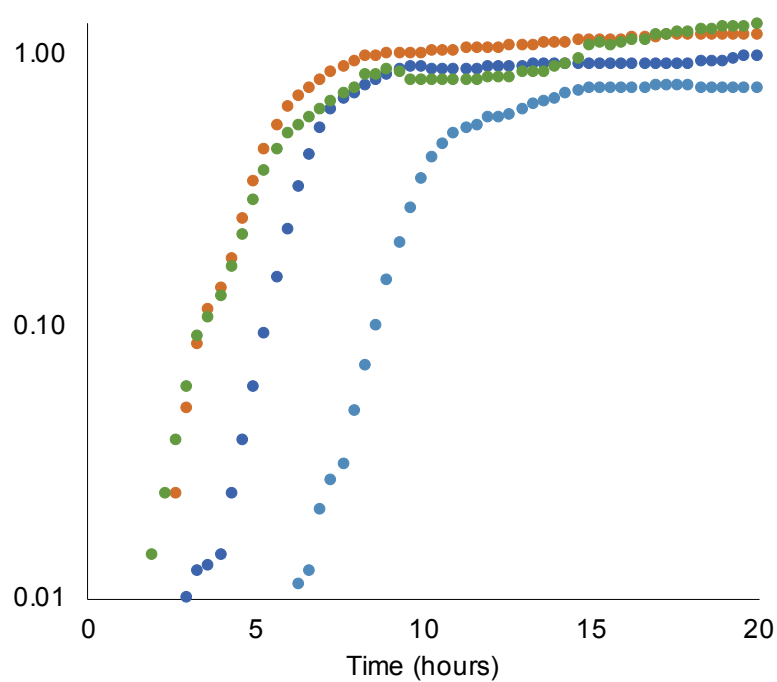

● WT - Daptomycin ● WT + Daptomycin ●  $\Delta ltaA$  - Daptomycin ●  $\Delta ltaA$  + Daptomycin

Supplement: S8 Fig — Growth curves for wild-type (WT) and ΔltaA strains for HG003 and MW2 in liquid media in the presence or absence of sub-MIC daptomycin at 37°C. For strains in the HG003 background, 2 μg/mL daptomycin was used; in the MW2 background, 1 μg/mL daptomycin was used. Each condition was replicated at least 3 times and a single growth curve representative of the trend is shown. (PDF) [file ppat.1007862.s008.pdf]
